# Intratumor heterogeneity of breast cancer detected by epialleles shows association with hypoxic microenvironment

**DOI:** 10.7150/thno.53737

**Published:** 2021-03-04

**Authors:** Yihan Wang, Yan Zhang, Yan Huang, Chuangeng Chen, Xingda Zhang, Ying Xing, Yue Gu, Mengyan Zhang, Li Cai, Shouping Xu, Baoqing Sun

**Affiliations:** 1 State Key Laboratory of Respiratory Disease, Guangzhou Medical University, Guangzhou, 51000, China.; 2 College of Bioinformatics Science and Technology, Harbin Medical University, Harbin, 150081, China.; 3 School of Life Science and Technology, Computational Biology Research Center, Harbin Institute of Technology, Harbin, Heilongjiang 150001, China.; 4 Department of Breast Surgery, Harbin Medical University Cancer Hospital, Harbin, Heilongjiang 150081, China.; 5 The Fourth Department of Medical Oncology, Harbin Medical University Cancer Hospital, 150 Haping Road, Harbin, 150040, China.

**Keywords:** breast cancer, intratumor DNA methylation heterogeneity, epiallele, methylation patterns, hypoxic microenvironment.

## Abstract

**Rationale:** In breast cancer, high intratumor DNA methylation heterogeneity can lead to drug-resistant, metastasis and poor prognosis of tumors, which increases the complexity of cancer diagnosis and treatment. However, most studies are limited to average DNA methylation level of individual CpGs and ignore heterogeneous DNA methylation patterns of cell subpopulations within the tumor. Thus, quantifying the variability in DNA methylation pattern in sequencing reads is valuable for understanding intratumor heterogeneity.

**Methods:** We performed Reduced Representation Bisulfite Sequencing and RNA sequencing for tumor core and tumor periphery regions within one breast tumor. By developing a method named “epialleJS” based on Jensen-Shannon divergence, we detected the differential epialleles between tumor core and tumor periphery (CPDEs). We then explored the correlation between intratumor methylation heterogeneity and hypoxic microenvironment in TCGA breast cancer cohort.

**Results:** More than 70% of CPDEs had higher epipolymorphism in tumor core than tumor periphery, and these CPDEs had lower methylation in tumor core. The CPDEs with lower methylation in tumor core may associate with hypoxic tumor microenvironment. Moreover, we identified a signature of five hypoxia-related DNA methylation markers which can predict the prognosis of breast cancer patients, including a CpG site cg15190451 in gene *SLC16A5*. Furthermore, immunohistochemical analysis confirmed that the expression of *SLC16A5* was associated with clinicopathological characteristics and survival of breast cancer patients.

**Conclusions:** The analysis of intratumor DNA methylation heterogeneity based on epialleles reveals that disordered methylation patterns in tumor core are associated with hypoxic microenvironment, which provides a framework for understanding biological heterogeneous behavior and guidance for developing effective treatment schemes for breast cancer patients.

## Introduction

As the most common diagnosed cancer in women, there will be approximately 62,930 new cases of female breast carcinoma *in situ* annually, which accounts for 30% of all new cancer diagnoses in women 1. The epigenome is at the intersection of the environment and genome. Epigenetic dysregulation, including DNA methylation, histone modification and miRNA alteration is important contributors of breast carcinogenesis. Recently, several studies have shown that epigenetics alterations associate with the development, diagnosis and therapy of breast cancer 2-4. Castelo-Branco et.al identified novel DNA methylation markers, of which cg12374721 (*PRAC2*)*,* cg18081940 (*TDRD10*) and cg04475027 (*TMEM132C*) could be useful for breast cancer classification and prognosis, particularly in estrogen-receptor (ER)-positive samples 5. A set of miRNAs modulated by diet and exercise were identified as diagnostic and prognostic biomarkers for breast cancer 6. The *MIR-127* and *MIR-125b-1* hypermethylation have been found to be novel biomarkers for breast cancer metastasis 7. As the potential for reversibility, epigenetic modifications are theoretically amenable to intervention and can be as a promising feature to optimize for devising novel therapeutic approaches. Recent studies have shown that promoter as well as intragenic and intergenic methylation widely modulate in tumor development and aggressiveness 8, 9. DNA methylation in promoters generally has a negative regulation effect of gene expression, whereas methylation in intragenic regions is not always associated with gene repression 10-12. Since the loss of gene body methylation might be also a contributing factor for the malignant cell state 13, we explored the DNA methylation changes in breast cancer at a variety of genome locations.

The imbalance of epigenetic regulation can also increase the plasticity of tumor cells 14, which is a crucial factor leading to tumor heterogeneity 15-17. Breast cancer is a highly complex heterogeneous disease at the molecular level, which forms different tumor subpopulations with distinct phenotypic characteristics 18, 19. The differences in DNA methylation pattern between different cell subpopulations can drive phenotypic changes, which is valuable for providing novel insights into the intratumor epigenetic heterogeneity of breast cancer. Although single cell bisulfite sequencing has the potential to make important contributions to the understanding of DNA methylation states of individual cells, the high costs and technical noise limit its applications. Alternatively, Reduced Representation Bisulfite Sequencing (RRBS) is an accurate and economical DNA methylation sequencing technique which can capture the probability distribution of DNA methylation patterns. As each read in the RRBS data can be viewed as a cell representation, epigenetic variations among cell subpopulations can be revealed by examining the frequency and distribution of different epigenetic allele patterns for all reads in one locus. Recently, the window-based measures for sequencing reads have been proposed, such as epipolymorphism 20, epigenetic allele (epiallele) 21, 22, and methylation haplotype blocks 23. Meanwhile, various scores have thus been proposed, such as Proportion of Discordant Reads (PDR) 24, Methylation Haplotype Load (MHL), Fraction of Discordant Read Pairs (FDRP) and quantitative FDRP (qFDRP) 25. Landan et al. indicated that the methylation status of a given locus in a cell population can be defined by a mixture of epialleles with variable frequencies. DNA methylation at consecutive CpG sites can establish a phase of epigenetic patterns (epialleles) that represent a "cellular barcode" of its own 20, 24. The study of epiallele can provide the dynamic evidences of differential methylation changes over time or by exposure to divergent environmental stimuli and facilitate better exploration of the intratumor heterogeneity of epigenome.

Here, we performed RRBS for multiple regions within one tumor to shed light on intratumor DNA methylation heterogeneity. We proposed a method “epialleJS” based on Jensen-Shannon divergence (JSD) to identify differential epialleles between tumor core and tumor periphery (CPDEs) and characterized tumor subpopulations with distinct methylation patterns. The methylation patterns of tumor core were more disordered than tumor periphery, suggesting a higher epigenetic heterogeneity. We also found that the genes with higher epigenetic heterogeneity also had higher transcriptional heterogeneity. Finally, we elucidated that the CPDEs with lower methylation in tumor core were linked to hypoxia, inferring hypoxic microenvironment can change the epigenetic states within the tumor. Methylation markers associated with hypoxic tumor microenvironment were also related to survival of breast cancer patients. Altogether, our study systematically analyzed the discrepancies of DNA methylation patterns within breast cancer cell subpopulations, which could help explain the causes and mechanisms of heterogeneity and provide precise personalized treatment protocols for breast cancer patients.

## Materials and Methods

### Clinical patients and samples

**Figure 1:**
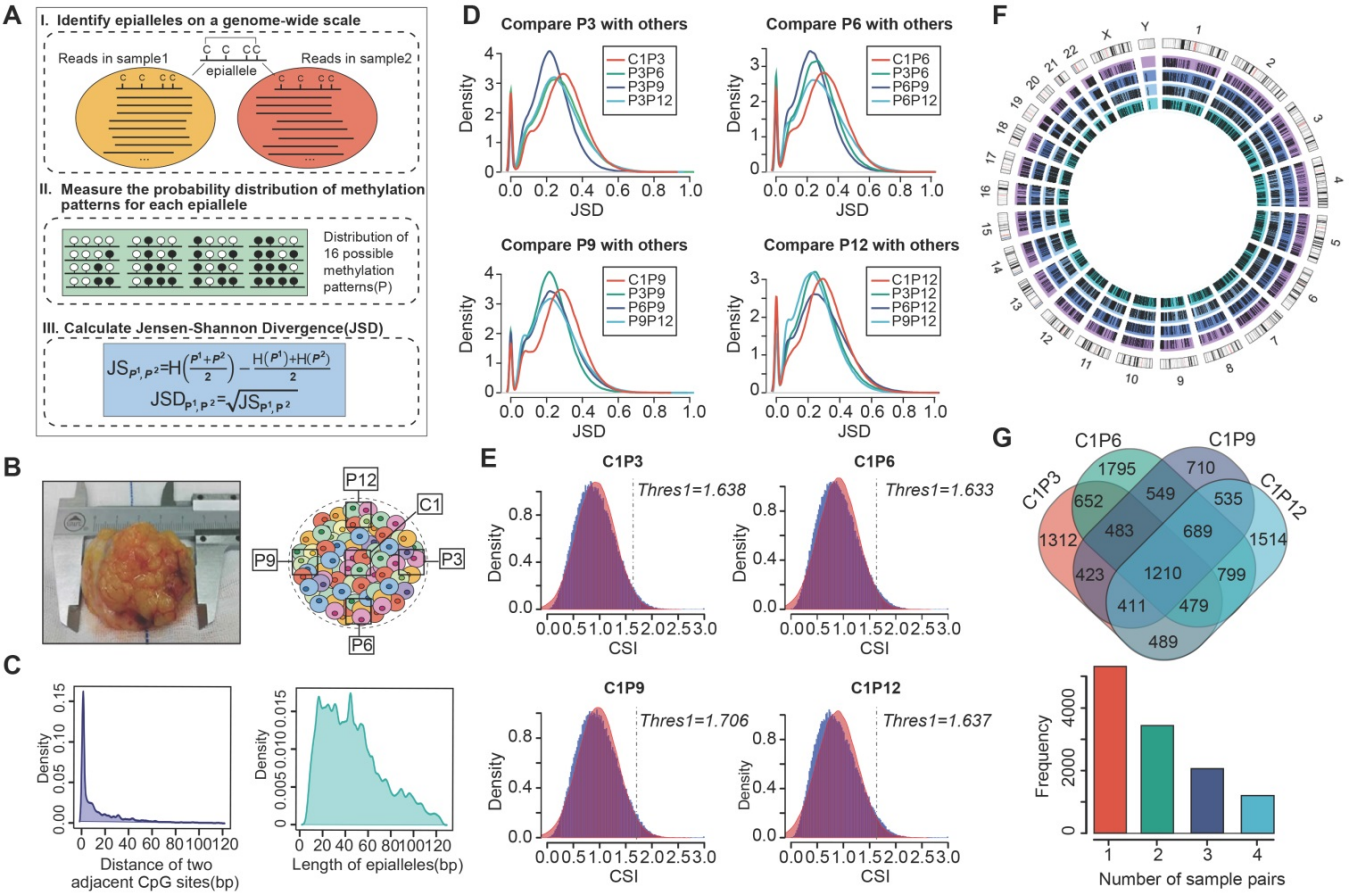
** The strategy for identifying differential epialleles between tumor core and tumor periphery (CPDEs).** (A) The workflow of “epialleJS” algorithm. (B)The tumor tissue from one patient and sampling points in tumor cross section, including one tumor core (C1) sample and four tumor periphery (P3, P6, P9 and P12) samples. (C) The probability density distributions of the distance between two adjacent CpG sites in epialleles covered by at least 10 reads and the length of epialleles. (D) The distributions of JSDs between one periphery sample and other tumor samples. (E) The distributions of CSIs and the thresholds for identifying CPDEs. Blue represents the real distribution of CSIs and red represents the normal distribution. (F) Positional distribution of differential epialleles between tumor core and tumor periphery (CPDEs). The tracks from inside to outside are CPDEs in C1P3, C1P6, C1P9 and C1P12. (G) The Venn diagram of four groups of CPDEs.

This study was approved by the Ethics Committee of Harbin Medical University, and the written informed consents were obtained from all participants prior to inclusion. Our tumor samples were derived from one patient with HER2 breast invasive ductal carcinoma at stage II. The size of tumor was 45 mm × 22 mm and the patient was without prior neoadjuvant therapy. For RNA and DNA libraries preparation, the tissues of tumor core, 12 o'clock, 3 o'clock, 6 o'clock and 9 o'clock of tumor periphery as well as adjacent of the tumor were taken for 0.6 mg, respectively (Figure 1B). Another case of normal breast tissue was used as control. In this study, 7 samples were examined both by RRBS and RNA sequencing.

### RRBS library preparation and sequencing

DNA was extracted from frozen tissue sections using the QIAamp DNA Mini Kit (QIAGEN GmbH, Germany) following the manufacturer's protocol. The tissue was ground and placed in a 2 ml centrifuge tube, 180 μl Buffer ATL was added. Vortexed for 20 s after the addition of 20 μl proteinase K at 56 ℃ until complete tissue lysis, and 200 μl Buffer AL was added and incubated in a 70 °C water bath for 10 min. We then added 200 μL of ethanol and put it into QIAamp Mini spin column for centrifugation at 6000×g for 1 minute. 500 μl Buffer AW2 was added to QIAamp Mini spin column and centrifuged at 20000×g for 3 min. The QIAamp Mini spin column was placed into a clean 1.5 ml collection tube, and centrifuged at 6000×g for 1 min at room temperature after adding 200 μl Buffer AE. Quantification was performed using a NanoDrop (Thermo Fisher Scientific) and checked for quality by agarose electrophoresis.

Library construction was conducted according to a previously published protocol 26. 5 μg DNA was diluted to 86 μl by adding RB and added 10 μl NEB4buffer and 4 μl MspI enzyme at 37 °C for 18 h. QIAquick PCR Purification Kit was used to purify the digested product according to the manufacturer's recommendations. 40 μl illuminaEndrepair buffer was added into 30 μl fragmented DNA, mixed and centrifuged. 160 ul AmpureBeads was added and mixed them thoroughly for 15 min at room temperature. 200 ul 80% ethanol was added and supernatant was removed. 20 ul RB was added and placed at room temperature for 5 min, and 17.5 ul supernatant was put into a new tube. Then 12.5 ul A-tailing buffer was added to 17.5 ul DNA at 37 °C for 30 min to perform end pair. 2.5 ul of Resuspension Buffer, 2.5 ul of Ligation Mix 2.5 ul ligation Mix, and 2.5 ul of the appropriate thawed DNA Adapter Index were added to each well of the ALP plate. 42.5 ul of mixed AMPure XP beads was add to each well of the ALP plate and incubated the ALP plate at room temperature for 15 min. Fragments of 150-175 bp or 175-225 bp were screened with 2% agarose gel electrophoresis and DNA was recovered by QIAquick Gel Extraction Kit. The DNA libraries were quantified using Qubit Instruments after PCR enrichment, and then were sequenced using a Hiseq2500 platform.

### RNA-seq library preparation and sequencing

Tissues were ground in liquid nitrogen, 1 ml TRIzol was added to 50-100 mg of the contents and mix thoroughly. The homogenate was incubated at room temperature for 5 min and centrifuged for 5 min at 12000×g at 4 °C. We transferred 1 ml of the supernatant to a new tube, added 200 ul chloroform (per ml Trizol), vortexed 15 s, incubated for 3 min at room temperature, and centrifuged for 15 min at 12000×g at 4 °C. Aqueous phase was transferred to a tube, and 500 ul isopropanol was added, vortexd and incubated for 30 min at room temperature. Then, the sample was centrifuged for 10 min at 12000×g at 4 °C and discarded all of the supernatant. The RNA precipitation was washed with 75% ethanol, 50-100 ul RNase-free water and 8M LiCl by half volume was added and placed on the ice for 1 h. Quantification was performed using a NanoDrop (Thermo Fisher Scientific) and checked for quality by agarose electrophoresis.

We performed dscDNA synthesis after RNA extraction, and then added 40 ul End Repair buffer to 60 ul fragmented DNAs for performing end repair. 12.5 ul A-tailing buffer was added to 17.5 ul DNA and PCR placed for 30 min at 37 °C. 2.5 ul Resuspension Buffe, 2.5 ul Ligation Mix, 2.5 ul the appropriate thawed DNA Adapter Index were added to each well of the ALP plate and incubated the ALP plate on the pre-heated thermal cycler, with the lid closed, at 30 °C for 10 min. The RNA libraries were quantified using Qubit Instruments after PCR enrichment and cluster generated using a Start cBot instrument, and then were sequenced using Hiseq2500 platform.

### Data processing

The 126 bp paired-end reads with an average depth of 25x for each covered CpG site were generated by RRBS (Table S1). Trim Galore was used to remove reads with poor quality and 5 '/ 3' end adapter sequences. The remaining reads were then aligned to the human GCRh37 / hg19 reference genome using the Bismark 27 and only the reads with unique alignments were analyzed. The mapped file with sam format for each sample was subsequently used for identifying epialleles. The 101bp paired-end reads data were generated by RNA-seq (Table S2). The raw reads were quality controlled using FastQC and then reads were aligned to the human GCRh37 / hg19 reference genome using Tophat2 28. The mapped reads were assembled into transcripts guided by Ensembl gene models using Cufflinks 29. To remove sources of bias in the data, the expression level of all transcripts was then normalized by Cuffnorm with default normalization method. The gene with mean FPKM greater than 1 was retained, and the fold change of the gene expression between the core sample versus each periphery sample was calculated, respectively. Finally, Cuffdiff was used to identify differentially expressed genes.

### Definition of the dissimilarity of epiallele

We defined four continuous CpGs covered by the same read as an epiallele. As the methylation status of a CpG was methylated or unmethylated, an epiallele contained 16 possible methylation patterns. We proposed a method named “epialleJS” relied on Jensen-Shannon divergence (JS divergence) 30 to quantify the dissimilarity between methylation patterns of two samples. The JS divergence of two methylation pattern probability distributions 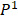 and 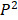 is defined to be


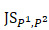
=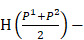

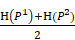
(1)

where 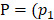,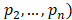, and 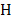 is the entropy of a probability distribution:


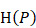
=
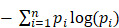
(2)


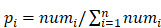
, 0
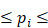
1;


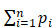
=1, and n=16

Where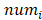 is the number of reads for pattern 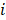 at a given epiallele, and 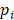 is the probability of pattern 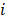. In order to avoid zero value in the antilogarithm, we added a small value 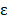 in the 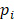 (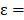
6.25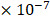). The entropy was then calculated as:


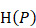
=
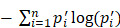
(3)


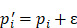


Relying on the theorem of Fuglede and Topsoe that JS is the square of a metric, thus, we define the dissimilarity of two probability distributions, 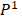
and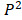 as


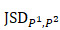
=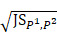
(4)


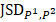
 is zero only when the distribution 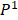 is identical with 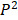, and is positive otherwise. Notably, JS divergence is a symmetric measure that is 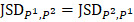. The open software “epialleJS” is available in GitHub repository (https://github.com/ccgBiotechLover/epialleJS).

### Detection of differential epialleles and local-specifc epialleles

To characterize intratumor DNA methylation heterogeneity, we identified differential epialleles between tumor periphery and tumor periphery (PPDEs), differential epialleles between tumor core and tumor periphery (CPDEs) as well as local-specific epialleles within the tumor. We required PPDEs to satisfy 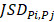
>δ and then determined the threshold δ by constructing a null hypothesis distribution of normal distribution and set the significance level α = 0.05 for one-tailed test.

The CPDEs were detected by considering both the dissimilarity between core and periphery and the dissimilarity between periphery and periphery for more reliable results. Thus, the composite specific index (CSI) for each periphery sample 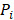 in each epiallele was defined. A higher CSI indicates a larger discrepancy between core and periphery compared to periphery and periphery.


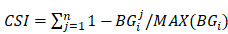
 and n=4 (5)

where 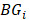
is the set of JSD between periphery sample 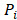 and other tumor samples (including one core sample and other three periphery samples)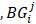 denotes the j-th element in the set 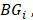 and 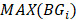 denotes the max element of set 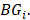 Differential epialleles between core sample *C*1 and periphery sample
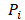
need to satisfy the following three requirements: (i) 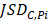
>γ; (ii) 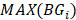
=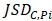; (iii) 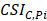
>*Thres1*. The value of γ was determined according to the distribution of JSD (here, γ=0.3). Then, after removing the consistent epialleles which JSDs in any two samples were 0, we calculated the CSI scores of epialleles for each pair of periphery sample and core sample. As the distribution of CSI scores followed an approximately normal distribution, a null hypothesis distribution of normal distribution was constructed and performed one-tailed test. The *Thres1* was determined by setting the significance level α = 0.05.

Local-specific epialleles within the tumor were detected by using the similar approach as above, including core-specific and each periphery-specific epialleles. The composite specific index (CSI) for each epiallele was defined. Here the CSI reflected the degree of specificity of the epiallele in a tumor sample.


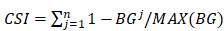
 , 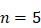
(6)


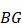

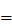
{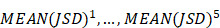}

where 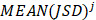 is used to denote the mean value of JSD between sample 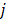 and the other tumor samples, and 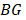
is the set of 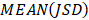 of all tumor samples. As we have five tumor samples, the set 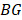 has five elements, and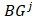 is the j-th element in the set 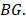 Similarly as above, specific epialleles for sample
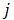
need to satisfy the following three requirements: (i)
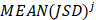
>γ; (ii)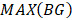 *=* 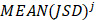; (iii)
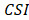
*>*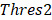. Here, we also set γ=0.3. A normal distribution was constructed and performed one-tailed test to obtain the *Thres2* by setting the significance level α = 0.05. The 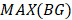 was used to assign the epiallele was specific to which tumor sample.

### Genomic annotations and functional enrichment analyses

Genomic annotations and positional information of functional elements were obtained from UCSC, and all genomic positions were based on the human genome sequence of Feb. 2009 (GRCh37 / hg19). The promoter region was defined as 1500 bp upstream of transcription start site (TSS) to 500 bp downstream of TSS. The CpG island shores were defined as 2kb regions at the left and right sides of the islands, and removed regions overlapped with CpG islands. The CpG island shelves were defined as 2kb regions at each side of the shores, and the regions overlapped with CpG islands and shores were removed. The positions of the repeating elements were obtained from Repeatmasker. Circos plots were drawn using Circos software 31.

GO and KEGG pathway enrichment analyses were performed using GREAT software 32. GREAT links genomic regions to genes by defining a regulatory domain for each gene. The region sets that included in the regulatory domain were used to calculate statistics by binomial and hypergeometric tests as a result of the enrichment.

### Epipolymorphism and methylation heterogeneity

The epipolymorphism of each epiallele was calculated based on Landan et al. 20. For one epiallele, the epipolymorphism was defined as

epipoly =1-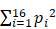
(7)

where 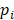 is the probability of epiallelic pattern* i*, and 16 possible patterns for the methylation status of four CpGs. Epipoplymorphism can be used to measure heterogeneity. The higher epipolymorphism indicates the higher epigenetic heterogeneity. Here, for each epiallele we compared its epipolymorphism changes between tumor core and tumor periphery. If epipolymorphism in the core sample was higher than that in the periphery sample, we defined the epiallele was a drift epiallele. On the contrary, we defined the epiallele was an adaptation epiallele.

Next, we further assessed the methylation heterogeneity of each sample based on epipolymorphism of CPDEs according to the method of Pan et al. 33. The average methylation levels of CPDEs were divided into 21 bins. The first bin was (0%, 2.5%), the last bin was (97.5%, 100%) and the width of remaining bins was 5. Calculating the median of epipolymorphisms of all the CPDEs in each bin as the epipolymorphism of this bin. The sum of the product of bin width and the median of its epipolymorphism was calculated as the area under the curve (AUC), which represents the methylation heterogeneity of this sample. The methylation heterogeneity ranges from 0 to 100.

### Determination of hypoxic state in TCGA breast cancer patients

We obtained four sets of breast cancer cell line expression data from GEO (GSE71401 34, GSE85353 35, GSE149132 36 and GSE111653 37) under hypoxic conditions and normal oxygen conditions. Differential expression analysis was performed with “edgeR” package on four datasets, respectively. Genes with FDR<0.05 and |log2FC|>=1 were defined as differentially expressed genes (DEGs). A total of 534 DEGs were selected as they were up-regulated under hypoxic condition in at least two data sets. In addition, 105 genes associated with hypoxia in breast cancer were collected from two literatures 38, 39. To classify TCGA breast cancer tumors into hypoxic and normoxic tumors, we performed unsupervised hierarchical clustering based Ward.D method on Z-score normalized RPKM for 19 overlapped genes that make up the hypoxia metagene signature (*ENO1*, *PYGL*, *PGK1*, *NDRG1*, *CA9*, *TPI1*, *VEGFA*, *PFKFB4*, *SLC2A1*, *P4HA1*, *LDHA*, *ADM*, *ALDOA*, *ANGPTL4*, *ADORA2B*, *PGAM1*, *BNIP3*, *COL4A5* and *KCTD11*). In the clustering, the top 2 sub-clusters identified were annotated as normoxic and hypoxic. The higher expression cluster was hypoxic cluster and the lower expression cluster was normoxic cluster.

### Unsupervised clustering of methylation levels of CpGs in TCGA breast cancer patients

The DNA methylation profile of breast cancer patients was downloaded from TCGA 450K platform and contained 684 samples. We only focused on the CpGs mapped to CPDEs. The methylation level of one CpG or the average methylation level of multiple CpGs in the same CPDE was used to represent the methylation level of the corresponding CPDE. By comparing of the methylation of CPDEs between tumor core and tumor periphery, the CpGs were divided into two sets and they were analyzed separately. Breast cancer samples were clustered using the Z-score normalized methylation levels of CpGs and they were classified into hypermethylation group, intermediate methylation group and hypomethylation group. Next, we analyzed the enrichment of hypoxic tumor samples in the hypermethylation group, intermediate group and hypomethylation group by using Cochran-Armitage Trend Test.

### Construction of hypoxic status prediction model

To test whether CPDEs can predict the hypoxic state of breast tumor, a random forest model was constructed by R packge “randomForest”. The inputs of the model were methylation levels of signatures and the output was hypoxic status (hypoxic or normoxic). Two-thirds of samples were randomly selected as the train set, and the remaining one-third of samples were used as the test set. For the train set samples, the accuracy of model was evaluated using the ten-fold cross-validation method.

### Survival analysis

The patients were randomly divided into a train set and a test set. Two sample sets were required to have the same size and clinical features had no significant difference. Univariate Cox regression and multivariate Cox regression were used to evaluate the association between prognosis and methylation level. In the multivariate cox regression analysis, CpG methylation, age and stage were used as covariates to exclude the contribution of clinical features. Then we assigned each patient a prognostic index (PI) according to a linear combination of the methylation levels of CpGs weighted by the regression coefficients from multivariate Cox regression analysis. The patients in the train set were divided into high-risk group and low-risk group by using the median PI as a cut-off point. According to the PI formula obtained by the train set, the PI values of patients in the test set were calculated, and they were divided into high risk group and low risk group using the same threshold as the train set. Kaplan-Meier plots were used to illustrate survival probability for high risk group and low risk group. The significance of differences in survival was estimated using the log-rank test. Survival analysis was carried out using the R package 'survival'.

### Immunohistochemical (IHC) staining

The breast cancer tissue specimens (n = 166) and normal tissue specimens (n=40) were purchased from Alenabio (www.alenabio.com; Xi'an, China), and IHC staining was performed according to the previous procedures 40. The protein expression level of SLC16A5 was assessed by IHC with the corresponding anti-MCT5 (dilution 1:50, ab180699, Abcam, Cambridge, MA, USA). The intensity of SLC16A5 staining was scored based on previously described criteria 40.

## Results

### Detection of differential epialleles between tumor core and tumor periphery (CPDEs)

Intratumor heterogeneity reflects potential cellular and molecular mechanisms of interaction between cancer cells and tumor microenvironment. The presence of tumor cell subpopulations makes a difference in the genetic, phenotypic and behavioral characteristics within the tumor or between metastatic parts. Here, we applied the concept of epigenetic alleles (epialleles) and proposed a method “epialleJS” based on the Jensen-Shannon divergence to explore the discrepancy between two samples (Figure 1A-B). The epialleles were required to be covered by at least 10 reads in each sample. Then, only the 663,009 epialleles shared in five tumor samples (background epialleles) were used for further analysis. In more than 80% of the background epialleles, the distance between two adjacent CpG sites was less than 40 bp (Figure 1C, left), and more than 80% of the epialleles had a length of less than 80 bp (Figure 1C, right).

Next, for each epiallele, we calculated the JSDs between any two tumor samples. It was found that the peak of JSDs between tumor core and tumor periphery were around 0.3 (red line), which were larger than that between different tumor peripheral samples (Figure 1D). Therefore, we set γ = 0.3. The distributions of CSI for each periphery sample and the core sample followed approximately normal distributions, and the CSI thresholds were determined by using one-tailed test, respectively (Figure 1E). Finally, we obtained 12,050 differential epialleles between tumor core and tumor periphery (CPDEs) in four sample pairs. Some CPDEs were located in oncogenes, such as *CCND1*, *KLF8*,* RUNX3* and *NOTCH1*. Specifically, P6 vs. C1 had the largest number of CPDEs (n=6656), and P9 vs. C1 had the lowest number of CPDEs (n=5150). Moreover, 5459 epialleles were differential between P3 and C1, and 6126 for P12 and C1 (Figure 1F). In fact, 44.24% (5331/12050) of the CPDEs were differential just in one sample pair and only 10% (1210/12050) of CPDEs were differential in four sample pairs (Figure 1G), suggesting that heterogeneity was also existed among different tumor peripheral regions. Thus, to further eliminate the heterogeneity between tumor core and tumor periphery was not caused by the differences between tumor periphery regions, we also detected differential epialleles between any of two periphery samples (PPDEs), and in total, 78099 PPDEs were identified (Figure S1).

### Characterization of compositional changes of CPDEs

**Figure 2:**
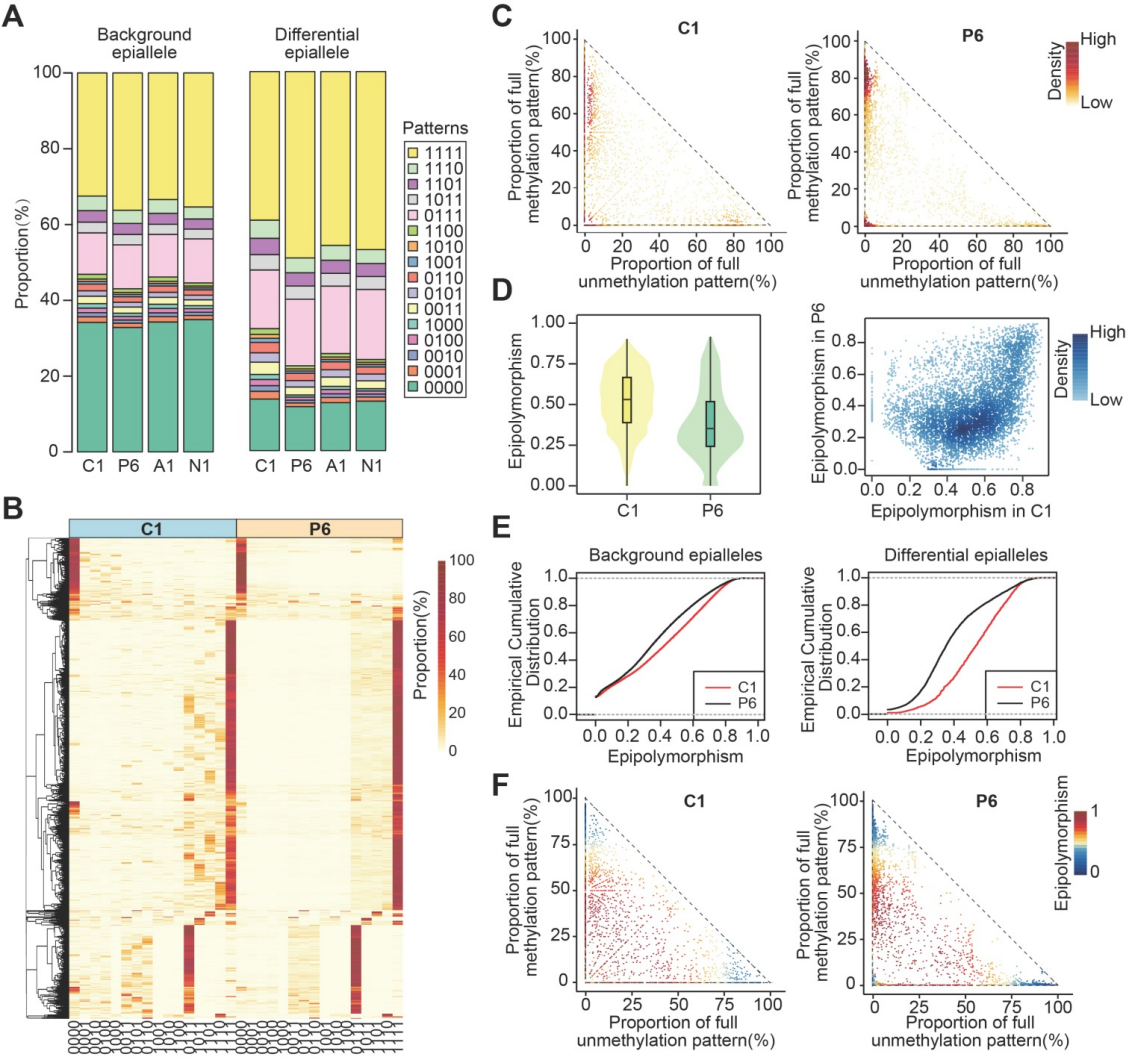
** The compositional changes and epipolymorphism of CPDEs.** (A) The composition of DNA methylation patterns in background and differential epialleles. Different colors represent all 16 methylation patterns. (B) The heatmap of compositional patterns of CPDEs identified between C1 and P6. (C) The distribution of full methylation ('1111') and full unmethylation ('0000') patterns in CPDEs identified between C1 and P6. Each point represents an epiallele, and the color represents the density of the point. (D) The epipolymorphism of CPDEs identified between C1 and P6 (Wilcoxon test, p<0.01) (E) The cumulative distribution of epipolymorphism of background epialleles and CPDEs identified between C1 and P6. (F) The epipolymorphism distribution of full methylation and full unmethylation patterns in CPDEs. Each point represents an epiallele, and the color represents the epipolymorphosim of epiallele.

Next, we compared the compositional changes of CPDEs with background epialleles. The composition of background epialleles was observed in the core sample (C1), periphery sample (P3/P6/P9/P12), adjacent sample (A1) and normal sample (N1) (Figure 2A and Figure S2). We used 16 colors to represent 16 patterns respectively, which '0' for unmethylated CpG sites and '1' for methylated CpG sites. The background epialleles were mainly composed of 6 DNA methylation patterns, including full unmethylation ('0000', average 34.06%), methylation at three sites (average total 20.46%; '0111', 11.36%; '1011', 2.71%; '1101', 2.86%; '1110', 3.53%), and full methylation ('1111', 34.38%) (Figure 2A), whereas the composition of CPDEs were changed compared with the background. For example, in the differential epialleles identified by C1 and P6, the proportion of full unmethylation pattern (average 12.72%) was reduced and the proportions of methylation at three sites and full methylation (average 28.67% and 45.29%, respectively) were increased, indicating the methylation patterns of regions with higher methylation level in breast cancer were more dynamic. To better display the compositional patterns, we showed the heatmap of the composition of each CPDE (Figure 2B and Figure S3). Consistent with the observation above that the major patterns were '0000', '0111' and '1111'. Meanwhile, the composition of each CPDE in tumor periphery was almost dominated by one pattern, representing a major cell subpopulation. Whereas it was diverse in tumor core, indicating that the cell subpopulations in tumor core was likely to be more various and heterogeneous. Moreover, we focused on the distribution of full methylation ('1111') and full unmethylation ('0000') patterns in CPDEs (Figure 2C). The CPDEs in tumor periphery were more likely to concentrate in the top left and the bottom left, implying a more obvious unimodal pattern in the high methylation level in tumor periphery.

Furthermore, we calculated the epipolymorphism of each epiallele, a measure that can reflect intratumor heterogeneity. The epipolymorphism value of an epiallele in a cell subpopulation was defined as the probability of epialleles by random sampling from different loci 20. The epipolymorphism of the core sample was higher, indicating that the core sample had more cell subclones with heterogeneous methylation patterns (Figure 2D and Figure S4, Wilcoxon test, p<0.01). The CPDEs were further divided into two categories, drift epialleles and adaption epialleles, and more than 70% of the CPDEs were drift epialleles. The cumulative distribution of epipolymorphism also showed that tumor core was more heterogeneous than tumor periphery (Figure 2E). As expected, the epialleles with full methylation (top left) and with full unmethylation (bottom right) had lower epipolymorphism, and the epialleles with multiple patterns (middle part) had higher epipolymorphism (Figure 2F).

### Methylation level and gene expression analysis for CPDEs

**Figure 3:**
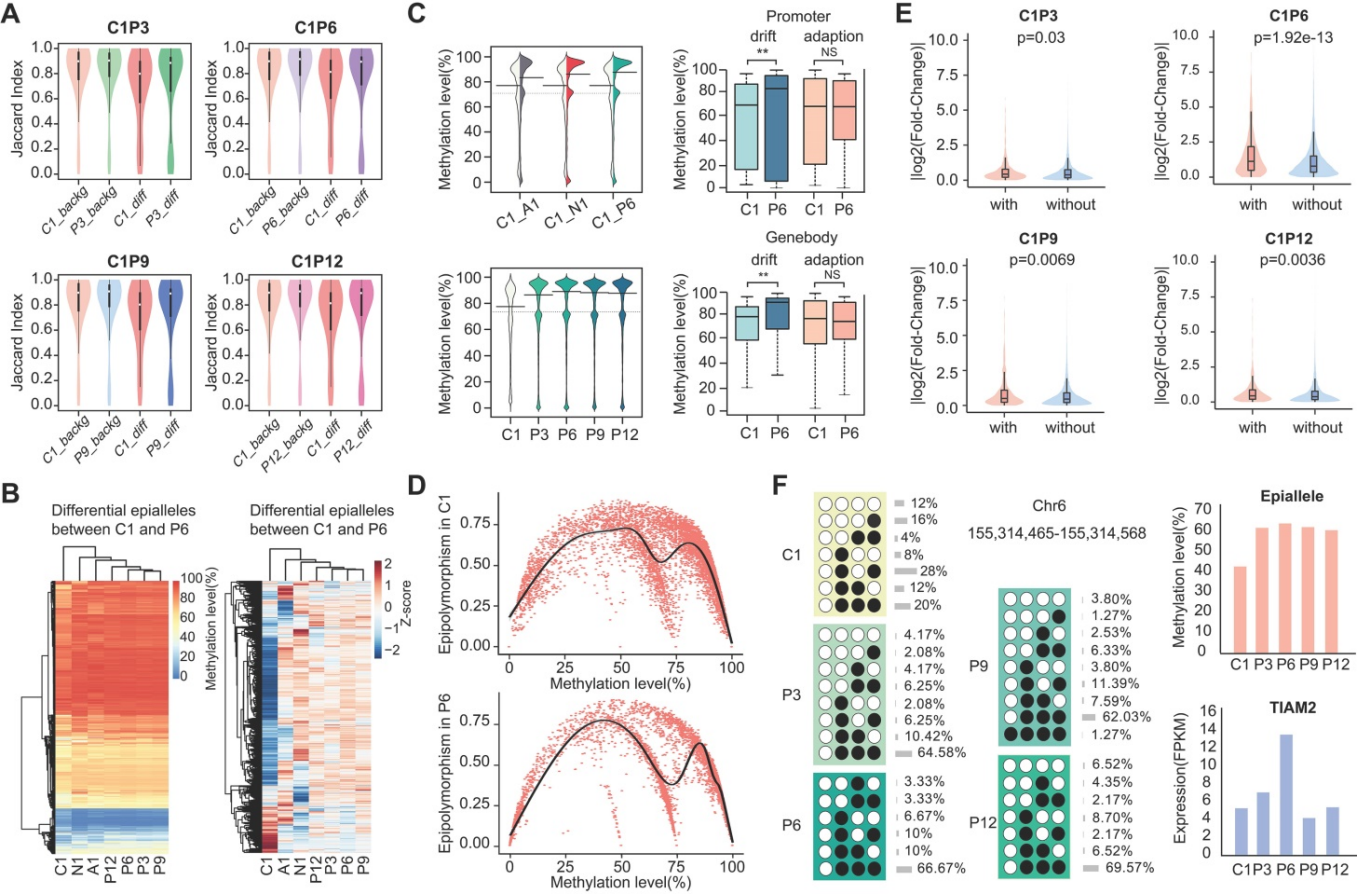
** The methylation and gene expression analyses for CPDEs.** (A) The Jaccard index of two adjacent CpGs within background epialleles and CPDEs, respectively. (B) The heatmap of raw methylation level and Z-score methylation level for CPDEs identified between C1 and P6. (C) The methylation levels of different genomic regions of CPDEs identified between C1 and P6. (D) The scatterplot of epipolymorphism corresponding to different methylation level in C1 and P6. (E) The violin plot of expression changes in genes which promoters with CPDEs and without CPDEs. The Y-axis represents log2 fold change of expression level of genes in C1 versus P6. In the X-axis, “with” represents genes with CPDEs in their promoters (red), and “without” represents genes without CPDEs in their promoters (blue). (F) An example of a CPDE located in *TIAM2*.

To explore whether differences in the epiallelic pattern of cellular subpopulations could lead to differences in average methylation level, we further investigated the methylation levels of CPDEs. Firstly, we calculated the similarity of methylation status between two adjacent CpG sites based on Jaccard index. The similarity in CPDEs was slightly lower than that in background, and the core sample was lower than the periphery sample (Figure 3A). Nonetheless, any two adjacent CpGs within the epialleles were highly correlated (r>0.8). Thus, it was feasible to use the average methylation level of the four CpG sites as the methylation level of this epiallele. It is noteworthy that compared to average methylation of all CpG sites within the CPDEs, average methylation of four CpG sites can better distinguish between tumor core and tumor periphery (Figure S5).

Next, we observed the raw and Z-score normalized methylation level. The methylation level was lower in tumor core than tumor periphery. Nevertheless, methylation levels of most CPDEs were high, which was consistent with that two major patterns '0111' and '1111 ' were found in CPDEs (Figure 3B and Figure S6). Moreover, the absolute differences in methylation levels of CPDEs and their JSDs were significantly positive correlated (Figure S7). Next, CPDEs were classified into four categories according to two changing models and genomic position. We found that the lower methylation level of tumor core was mainly affected by drift CPDEs, which accounted for more than 70% of all CPDEs. For drift CPDEs, methylation levels were differential both in promoter and gene body (Figure 3C and Figure S8). Since the dynamics of epiallelic patterns can affect methylation level, we calculated the methylation heterogeneity among tumor samples in CPDEs according to Pan's method 33 (Figure 3D). Finally, the AUC of tumor core was found to be greater than tumor periphery in all sample pairs, indicating that tumor core had higher methylation heterogeneity than tumor periphery (Figure S9). We hypothesized that the microenvironment in tumor core regions might led to more diversification of tumor subclones.

It has been reported that local disordered methylation is associated with gene expression 24. Here, we explored whether the intratumor heterogeneity detected based on epialleles was also associated with gene expression changes. The results showed a significant difference between the promoters with CPDEs and without CPDEs (Figure 3E) (Wilcoxon rank sum test, C1P3, p=0.03; C1P6, p=1.92e-13; C1P9, p=6.94e-3; C1P12, p=3.60e-3). Moreover, there was a higher proportion of differentially expressed genes in genes with CPDEs in their promoters (C1 vs. P3, 3.10% vs. 2.81%, OR = 1.10; C1 vs. P6, 36.44% vs. 18.78%, OR = 1.94; C1 vs. P9, 7.19% vs. 3.68%, OR = 1.95; C1 vs. P12, 5.38% vs. 3.15%, OR = 1.71). Taken together, it was indicated that genes with higher epiallelic pattern heterogeneity also had higher transcriptional heterogeneity. To more intuitively understand the concept of epiallele and the changes of methylation level caused by differential epialleles, we showed an example of epiallele chr6:155314465-155314568, locating in the promoter of *TIAM2*. It was a differential epiallele between the core sample and all peripheral samples, and the methylation levels varied greatly between them (Figure 3F).

### Genomic distribution and functional analysis of CPDEs

**Figure 4:**
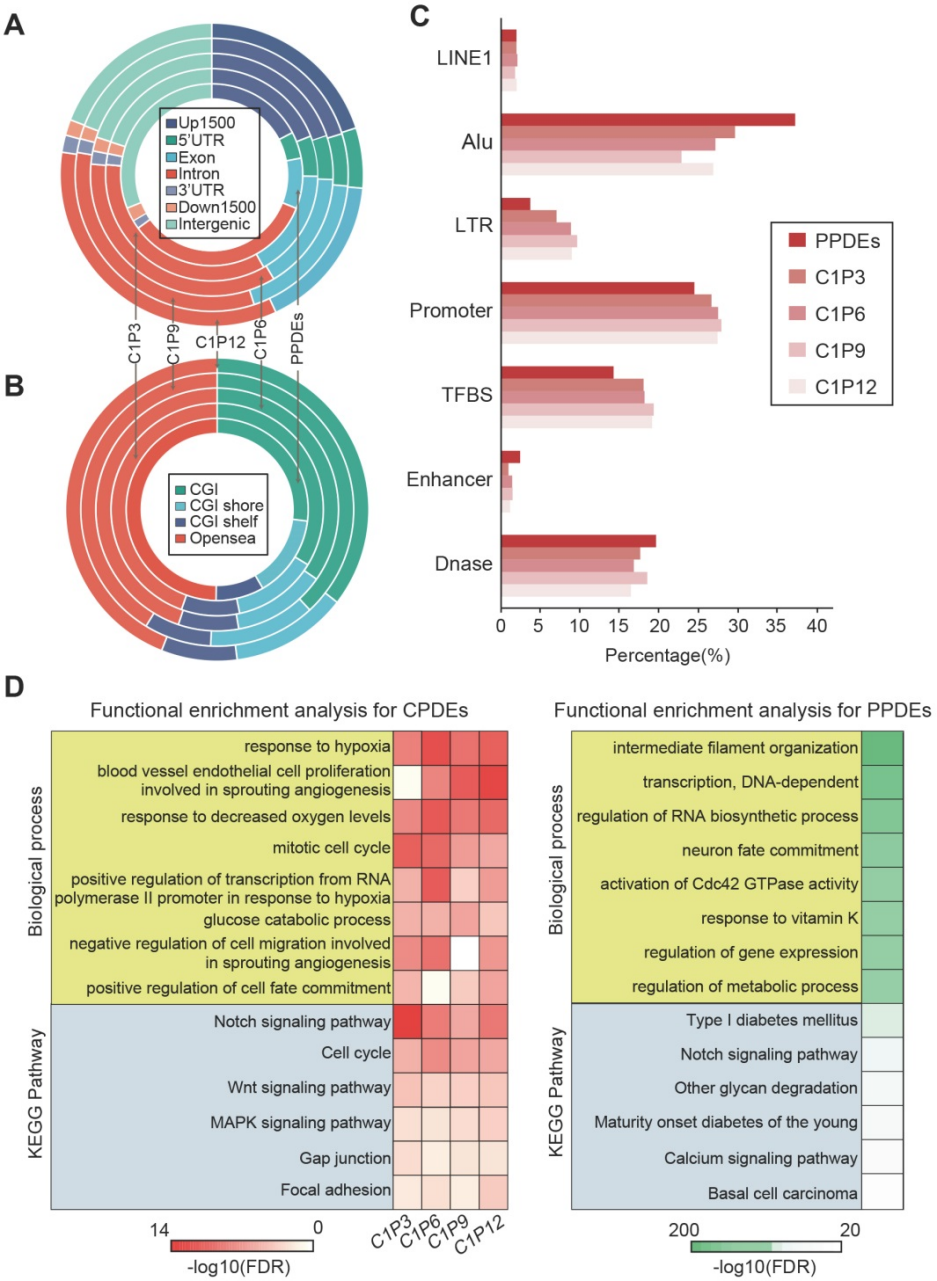
** Genomic distribution and functional analysis.** (A) Distribution of genomic locations for CPDEs and PPDEs. (B) Distribution of CGIs, CGI shores, CGI shelves and opensea for CPDEs and PPDEs. (C) The Overlap of CPDEs and PPDEs with known functional elements. (D) The GO biological process and KEGG pathway enrichment analyses for CPDEs and PPDEs.

CPDEs were widespread events in the human genome. To elucidate this, we used the Ensembl gene model to observe the distribution of CPDEs in their most recent genes (Figure 4A). An average of 5813 CPDEs were obtained from each pair of tumor core sample and tumor peripheral sample. It was revealed that only 19.6% (1138/5813) of CPDEs were located in intergenic regions. And 80.4% (4675/5813) of CPDEs were located in transcriptional regions, including 25.9% (1505/5813) in promoter regions (Up1500 and 5'UTR) and 51.1% (2970/5813) in gene body regions (exon and intron). In addition, since there were epigenetic differences between different tumor peripheral regions, we investigated the genomic distributions of PPDEs. Compared to CPDEs, PPDEs were more likely to be located in intergenic regions (31%, 24179/78099) and the ratio in promoter was decreased (22.1%, 17225/78099). Next, we examined the region-specific enrichment of CPDEs and PPDEs relative to CpG density (Figure 4B). We observed that 35.5% (2059/5813) of CPDEs were located in CpG islands (CGIs), 12.6% (731/5813) in CGI shores, 8.3% (480/5813) in CGI shelves and 43.6% (2543/5813) in opensea. Whereas for PPDEs, the ratio in CGIs (27%, 21105/78099) was decreased and the ratio in opensea was increased (49.8%, 38898/78099). Further, the overlaps between CPDEs, PPDEs and known functional elements were observed, respectively (Figure 4C). We found that the ratios of CPDEs overlapped with TFBS and promoter were higher than PPDEs, and the ratios of CPDEs overlapped with DNase and enhancer were lower than PPDEs, which indicated that intratumor heterogeneity was associated with transcriptional regulation, and the CPDEs captured more direct characteristics of transcriptional regulation. Moreover, CPDEs were more likely to be located in LTR and PPDEs were in Alu.

The DNA methylation pattern of epialleles describes a novel characteristic which is distinct from a single CpG. We used GREAT software to perform enrichment analyses of GO biological processes and KEGG pathways for the CPDEs and PPDEs, respectively. The CPDEs were enriched in response to hypoxia, negative regulation of cell migration involved in sprouting angiogenesis, cell cycle phase, glucose catabolic process and positive regulation of cell fate commitment, and enriched in pathways such as Notch signaling pathway, Wnt signaling pathway and MAPK signaling pathway (Figure 4D). The enrichments indicated that these changes in methylation patterns contributed to disrupted pathways in the progression of breast cancer. Notably, we found that intratumor heterogeneity was related to hypoxia. While the PPDEs were enriched in intermediate filament organization, transcription, regulation of RNA biosynthetic process, regulation of metabolic process, and the pathways such as Type I diabetes mellitus, Notch signaling pathway and calcium signaling pathway, which shed light the different roles of CPDEs and PPDEs played in tumor occurrence and progression.

### Detection of local-specific epialleles for different regions within the tumor

**Figure 5:**
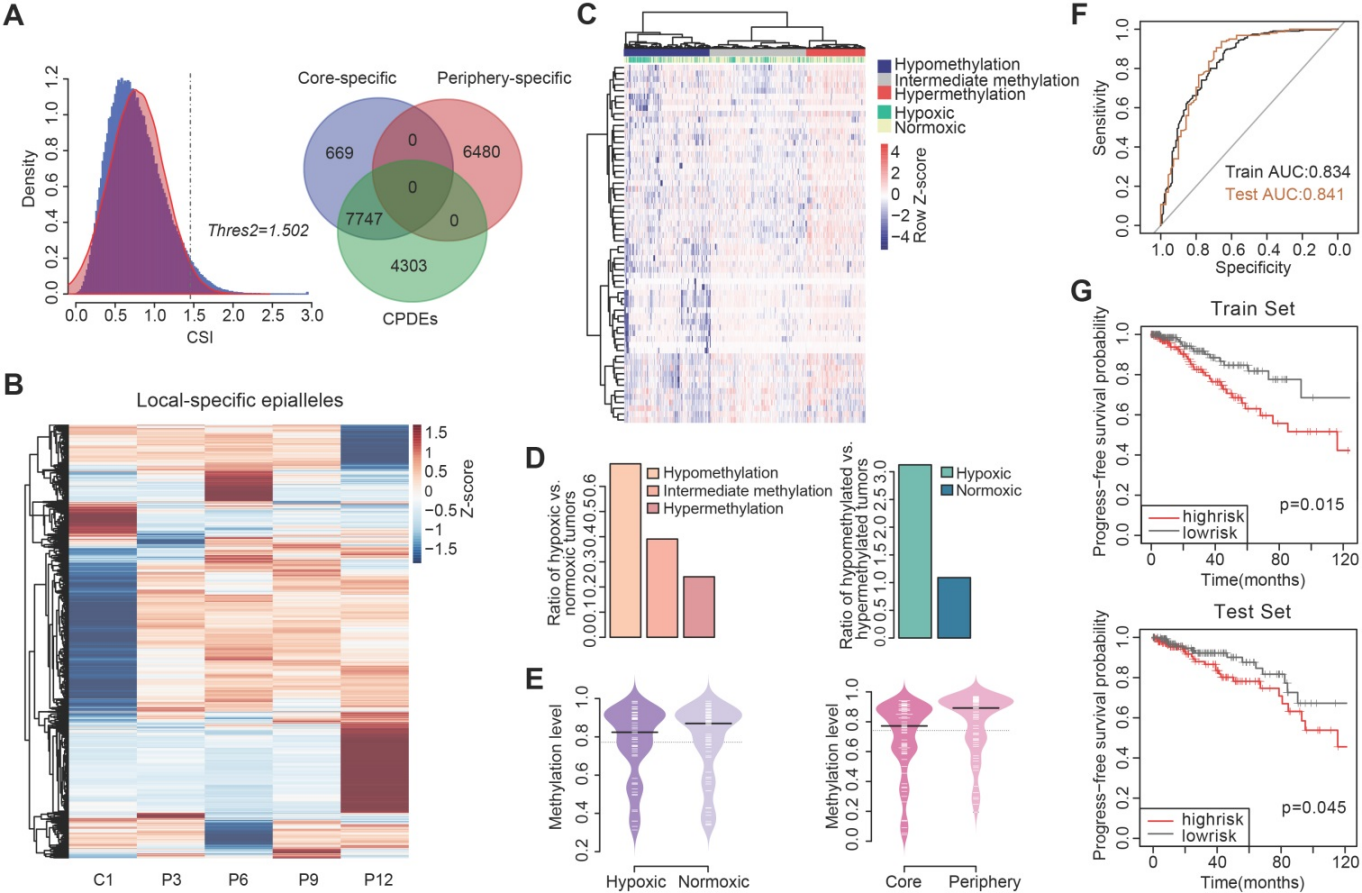
** The identification of local-specific epialleles and hypoxia analysis combined with TCGA BRCA methylation profiles.** (A) The identification of tumor periphery-specific epialleles. (B) The heatmap of methylation level for tumor core- and tumor periphery-specific epialleles. (C) The hierarchical clustering for TCGA BRCA patients based on methylation of CPDEs with lower methylation in tumor core than tumor periphery. (D) Ratios of hypoxic tumors vs normoxic tumors in the hypomethylation, intermediate methylation and hypermethylation groups (Cochran-Armitage trend chi-square test, p=2.314e-06), and ratios of hypomethylated tumors vs hypermethylated tumor in the hypoxic and normoxic clusters (chi-square test, p=8.789e-06). (E) Comparison of methylation level in hypoxic and normoxic clusters, as well as methylation level in tumor core and tumor periphery. (F) ROC curves for random forest predict models (The black line represents the train set and the brown line represents the test set). (G) The progression-free survival (PFS) analysis in the train set and test set (Log-rank test p=0.015 for train set and p=0.045 for test set).

As communication with distinct local tumor microenvironment allows the diversity of cell subpopulations within the tumor, we also examined the local-specific epialleles including core-specific and periphery-specific epialleles. Here, the threshold of γ was 0.3 and the CSI distribution of all epialleles followed an approximately normal distribution (Figure 5A). We obtained 14896 local-specific epialleles, including 8416 core-specific (C1) and 6480 periphery-specific epialleles (253 for P3, 1742 for P6, 93 for P9 and 4392 for P12). Interestingly, 92.05% (7747/8446) of core-specific epialleles were CPDEs, while there was no overlap between periphery-specific epialleles and CPDEs, which indicated that CPDEs mainly reflected the characteristics of tumor core. Similarly, the specificity in methylation levels of these local-specific epialleles was also observed from the heatmap, especially for samples with more specific epialleles, such as C1, P6 and P12 (Figure 5B).

Functional enrichment analysis of two types of specific epialleles revealed that core-specific epialleles enriched in mitotic telophase and anaphase, negative regulation of Notch pathway, response to hypoxia and response to decreased oxygen levels. While periphery-specific epialleles involved in negative regulation of nitric-oxide synthase activity, negative regulation of adenylate cyclase activity, negative regulation of cAMP biosynthetic process and negative regulation of oxidoreductase activity. These results indicated that core-specific epialleles mainly involved in cell division and hypoxia-related biological processes, and periphery-specific epialleles mainly involved in the biological processes related to signal transduction.

### Intratumor heterogeneity is linked to hypoxic tumor microenvironment

As tumor cells proliferate indefinitely, insufficient oxygen supply renders a hypoxic microenvironment in the tumor core. Hypoxia is the driver of genetic instability and can induce changes in epigenetic states of tumor cells, leading to more aggressive tumor phenotypes. Thus, hypoxia could be involved in the formation of intratumor heterogeneity to promote tumor adaptation and evolution. In the functional analysis, we found that CPDEs were more enriched in hypoxia-related functions than PPDEs, indicating that epigenetic regulation within the tumor was affected by the hypoxic microenvironment. Here, we focused on CPDEs and inferred the cells in tumor core were more likely to be hypoxic than periphery cells. To assess whether hypoxia contributes to the formation of intratumor heterogeneity and identify novel hypoxia-associated biomarkers, we incorporated data from breast cancer cell lines in GEO and breast cancer methylation profiles and clinical information in TCGA for further analysis.

Firstly, the hypoxic status of breast cancer patients in TCGA was determined by integrating differentially expressed genes under hypoxic and normoxic conditions and hypoxia-associated genes derived from literatures (Figure S10). Then, to explore whether the differential methylation patterns between tumor core and periphery were related to tumor hypoxia, we combined 1210 CPDEs which were differential in four sample pairs with 450K methylation profile of TCGA breast cancer patients for subsequent analysis. Of the 1210 CPDEs, only 97 CPDEs contained 83 CpG probes. 68 CPDEs with lower methylation in tumor core than periphery contained 62 CpGs, and 29 CPDEs with higher methylation in tumor core than periphery corresponded to 21 CpGs (Figure S11).

The two sets of CpGs were used to cluster breast cancer patients respectively. Firstly, we analyzed the enrichment of hypoxic tumor samples in the hypermethylation, intermediate methylation and hypomethylation groups based on the set of 62 CpG sites with lower methylation in tumor core (Figure 5C). The proportion of hypoxic tumors decreased with increasing methylation levels (Cochran-Armitage trend chi-square test, p=2.314e-06), and hypoxic tumors were more prone to lower methylation (chi-square test, p=8.789e-06) (Figure 5D and Table S3). In addition, we further compared the methylation levels of corresponding 68 CPDEs in the hypoxic and normoxic clusters. The result showed that the methylation levels of hypoxic cluster were lower than that of normoxic cluster. Moreover, the methylation levels of tumor core were more like that of hypoxic cluster, and the methylation levels of tumor periphery were closer to that of normoxic cluster, indicating that cells in tumor core were more likely to be under the hypoxic microenvironment (Figure 5E). The CPDEs with lower methylation in tumor core than tumor periphery played an important role in the formation of tumor heterogeneity (Table S4). For example, extracellular superoxide dismutase (*SOD3*) is a secretase that regulates the balance of redox reactions in tissues and regulates tumor vascular distribution in tumors, increasing the sensitivity of tumors response to chemotherapy 41. BTG3-associated nuclear protein (*BANP*) can form a compound with *p53* and negatively regulate *p53* transcription, and *BANP* is a tumor suppressor which can regulate cell cycle.

However, when the same analysis was performed using the set of 21 CpG sites, there was no obvious linear trend between the proportion of hypoxic tumors and the changes in methylation level (Figure S12A). The methylation patterns of the hypoxic and normoxic clusters were different from that of tumor core and tumor periphery (Figure S12B), indicating that the higher methylation level in tumor core than tumor periphery may not be affected by hypoxia.

### CPDEs can predict hypoxic status and associated with prognosis of breast cancer patients

To further examine whether the CPDEs with lower methylation in tumor core than tumor periphery had the ability to predict tumor hypoxia status, we identified 34 differentially methylated CpGs between hypoxic cluster and normoxic cluster (wilcoxon rank sum test, p < 0.05) and then used them to construct a random forest model. The accurate rate was 82.2% and the area under the curve (AUC) was 0.834 in the train set (Figure 5F). Applying the model to the test set, the prediction accuracy rate was 84.3% and the AUC was 0.841, implying that the model constructed by these CpGs had a well predictive performance on hypoxic status. They can be considered as new hypoxia-related DNA methylation markers. Using the MeanDecreasedGini changes as the importance measure of the input features, the top five important features were cg00409636, cg15190451, cg11339839, cg04848343 and cg08387141. The CpG site cg00409636 is located on *FAM176A* which regulates programmed cell death and mediates autophagy and apoptosis. The CpG site cg04848343 is located on *SEMA6B* which is involved in cell differentiation and axon guidance pathways and is an important member of the axon guidance factor family. This family plays a major role in tumor development and cell migration. Studies have shown that *SEMA6B* promotes angiogenesis through the Rho kinase signaling pathway 42. *SEMA6B* is also associated with tumor differentiation, lymph node metastasis and distant metastasis in gastric cancer 43.

We then performed survival analysis on hypoxia-associated markers and the results showed that cg15190451, cg08120511, cg27413290, cg10169763 and cg15891447 were associated with progression-free survival of patients after exclusion of clinical factors (p<0.05), and their HRs were all less than 1 (Table 1), revealing that the risk of breast cancer progression increased with the decrease of methylation level. Using these 5 CpGs as a methylation feature, a risk scoring system was constructed to calculate the risk score PI for each patient: PI=(-1.875×cg15190451) + (-1.669×cg08120511) + (-2.307×cg27413290) + (-5.451×cg10169763) + (-2.51×cg15891447). The median of PI (-11.1) was taken as a threshold to divide the patients from the train set into two groups. The results showed that patients in high risk group had worse survival than those in low risk group (Figure 5G, p=0.015, log-rank test). Similarly, the high risk group in the test set also had worse survival than the low risk group (Figure 5G, p=0.045, log-rank test).

### Association of SLC16A5 with clinicopathological characteristics and prognosis in patients with breast cancer

**Figure 6:**
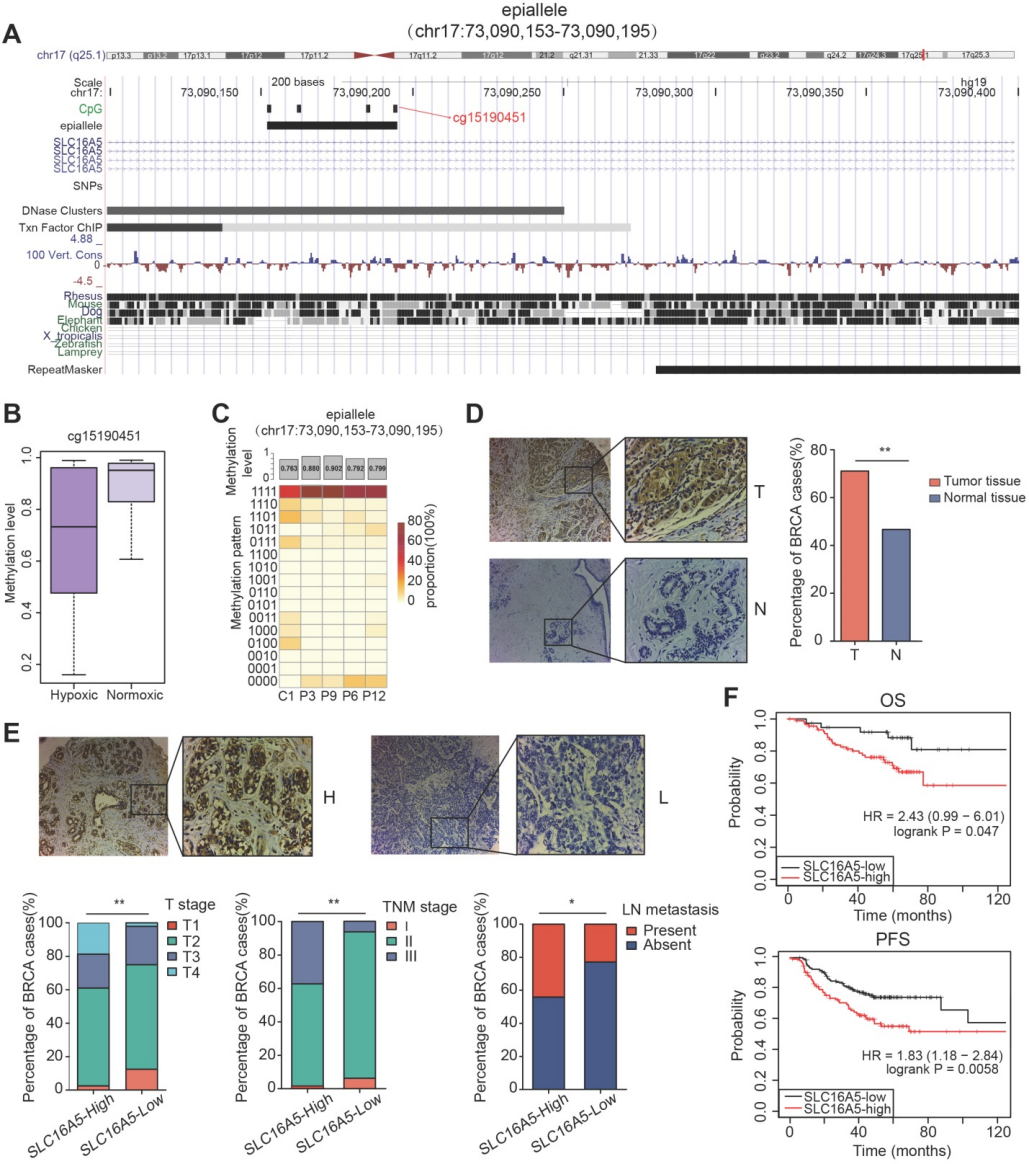
** Methylation analysis of the epiallele in *SLC16A5* and immunohistochemistry analysis of *SLC16A5*.** (A) UCSC browser visualization of the positions of cg15190451 and its corresponding epiallele chr17:73090153-73090195. (B) The boxplot of methylation level of cg15190451 in hypoxic cluster and normoxic cluster for TCGA BRCA samples (Wilcoxon test, p=3.778e-16). (C) The methylation level and methylation patterns of the epiallele in tumor core (C1) and tumor periphery (P3, P6, P9 and P12). (D) Immunohistochemistry images of breast tumor (T) and adjacent normal breast tissue samples (N). Significantly darker brown staining of *SLC16A5* protein was detected in cancer tissues than in adjacent normal tissues. The percentage of breast tissues with high *SLC16A5* expression was significantly greater than that of normal tissues, **p < 0.01 by chi-square test. (E) Immunohistochemistry images of *SLC16A5*-high expression samples (H) and *SLC16A5*-low expression samples (L). * p < 0.05, **p < 0.01 by chi-square test. (F) Kaplan-Meier curves of OS and PFS based on HER2-positive breast cancer patients.

Interestingly, in the five prognosis-associated CpG sites, we found that the CpG site cg15190451 was also included in the top five important features of random forest prediction model described above. The CpG site cg15190451 was mapped to epiallele chr17:73090153-73090195, locating on gene *SLC16A5* (Figure 6A). The methylation level of cg15190451 was lower in hypoxic tumors than normoxic tumors (Figure 6B, Wilcoxon test, p=3.778e-16). Meanwhile, the epiallele had more disordered methylation patterns and lower methylation in tumor core than tumor periphery (Figure 6C). *SLC16A5* is a member of the monocarboxylic acid transporter family which plays an important regulatory role in tumor cell energy metabolism and tumor microenvironment. When cells are under hypoxic conditions, lactic acid is produced by the glycolysis process. At normal physiological pH, lactic acid does not pass freely through the cytoplasmic membrane, relying mainly on the transport between cells by the monocarboxylate transporter. If lactic acid cannot be transported in time and accumulates in the cell, it will cause a decrease of intracellular pH value, leading to cell apoptosis. Therefore, monocarboxylate transporters are critical for the survival and metastasis of tumor cells under hypoxic conditions.

Next, we further evaluated the expression of *SLC16A5* in 166 breast cancer tissues and 40 normal tissues by immunohistochemical staining. The results showed that *SLC16A5* expression was significantly higher in tumor tissues than that in adjacent tissues (chi-square test, p < 0.01) (Figure 6D). In addition, immunohistochemical analysis confirmed that high expression of *SLC16A5* was associated with larger tumor size, lymph node metastasis, and advanced TNM stage in breast cancer (Figure 6E). The Kaplan-Meier analysis revealed that high expression of *SLC16A5* was correlated with poor prognosis of both OS and PFS in HER2-positive breast cancer patients (p < 0.05, Figure 6F).

## Discussion

DNA methylation as a regulator of gene expression plays a critical role in normal growth and breast tumor development 44. The hypermethylation of tumor suppressor genes results in uncontrolled growth of tumor cells, whereas global hypomethylation tends to genomic instability and activation of oncogenes 45. They have been reported as promising diagnostic and prognostic biomarkers, or potentially efficient therapeutic targets for breast cancer 5, 6, 46. DNA methylation changes mainly originate from differences between cells and cells. Thus, understanding the diversity of DNA methylation patterns across cell subpopulations can provide important additional information about intratumor heterogeneity. However, most current studies are based on single CpG site methylation, which is limited by the technical noise and sensitivity of detection. Compared with single CpG site, the analysis of epialleles can reveal the dynamics of methylation status and it is suitable for studies with small sample size. By detecting the diverse patterns of epialleles, it can discover the characteristics of different cell subpopulations and quantify intratumor heterogeneity. Moreover, epiallelic DNA methylation pattern provides complementary information to DNA methylation level. For example, some of the epialleles had large JSDs between tumor core and tumor periphery, but the differences of average methylation levels were small (such as epiallele chr19:1299820-1299851, in the exon of gene *EFNA2*; for C1 and P6, JSD=0.687, meth_C1=0.725, meth_P6=0.823).

Here, we first obtained the probability distributions of all 16 patterns for an epiallele shared in two samples, and then defined the dissimilarity of these two distributions based on Jensen-Shannon divergence. The range of Jensen-Shannon divergence is from 0 to 1, so it is convenient for the comparison between different sample pairs. Then we constructed a comprehensive specificity index (CSI) for each of shared epialleles to identify differential epialleles and local-specific epialleles. When defining the differences between tumor core and periphery, in order to make the results more rigorous, we considered not only the dissimilarity between tumor core and tumor periphery, but also the dissimilarity between periphery samples. The dissimilarity between tumor core and tumor periphery should larger than that between periphery samples. When defining specificity, we improved the measure of previous specificity identification. Most previous methods were based on the measure of itself, while our method used the dissimilarity between two samples (JSD) for specificity identification.

The functional analysis of CPDEs revealed that they were involved in several carcinogenic and hypoxia-related biological processes. Hypoxia is an important factor affecting the diversity of tumor cells, and adaptation to hypoxia is a key step in tumor survival and development. Hypoxic tumor microenvironment is closely related to tumor angiogenesis, oxygen supply and energy metabolism mechanisms. The self-regulation and adaptation of tumor cells to ischemia and hypoxia are mainly through the enhancement of glucose transport, glycolysis and tumor angiogenesis 47. In fact, most of the malignant tumors have internal hypoxic regions in their growth and development processes, which mainly due to the rapid expansion of the tumor volume. Parts of the tumor tissue are more distant from the nearest blood vessels to lead to a lack of blood supply and hypoxia. Tumor hypoxia will cause its tolerance to chemotherapy and radiotherapy and increase tumor metastasis, while hypoxic microenvironment is an important feature of the formation of tumor heterogeneity 48. The heterogeneity of tumor microenvironment determines the adaptability of the tumor and may therefore be a key factor in the success of the treatment 49. Now some targeted therapies for hypoxia 50, 51 or epigenetics 52, 53 have been successfully applied in clinical and found that hypoxia-driven pathways can be attenuated by supplemental oxygen to promote tumor regression 54.

In addition, we have demonstrated in many aspects that the DNA methylation heterogeneity in tumor core is stronger, such as more disordered epiallelic patterns, higher proportion of drift CPDEs, and larger AUC in tumor core. In an earlier study, they proposed that hypoxia as a strong evolutionary selection pressure can lead to a variety of metabolic phenotype of cancer 55. The tumor cells under hypoxic microenvironment were regulated by various cellular mechanisms to enable their phenotypes switching to multiple forms for adapting this unfavorable environment, which may explain the higher heterogeneity in tumor core. And the hypoxic tumor cells were more resistant and survivable, and developed to malignant phenotype. Further we showed that the changes in average methylation level can be explained by epiallelic diversity, and the genes with higher epiallelic heterogeneity had higher transcriptional heterogeneity. Finally, we identified novel epigenetic markers associated with hypoxic tumor microenvironment. They can be used to classify the hypoxic status of breast cancer patients and associated with progression-free survival, suggesting that the presence of hypoxia affected the patient's metastasis and relapse. A study has shown that in invasive diseases, tumor hypoxia is likely to be a strong predictor for metastasis 56.

However, our study had several limitations. As DNA methylation is affected by environmental and individual factors, we analyzed intratumor DNA methylation heterogeneity from only one breast cancer patient without considering the inter-individual heterogeneity and subtype difference, the findings revealed here may not be comprehensive enough. It will be conducive to explore tumor evolution if different regions in multiple tumors can be detected in future analysis. Besides, further works will focus on exploring whether the quantification of disorders at the level of the epialleles instead of average methylation level at four sites can provide a more accurate measure of tumor aggressiveness.

Tumor heterogeneity poses major challenges for diagnosis and treatment in clinical. The infinite proliferation of tumor cells and even metastasis are not only caused by changes in molecular level, but also tumor microenvironment plays an essential regulatory role in the tumor. Intratumor heterogeneity reflects the underlying cellular and molecular mechanisms of interaction between tumor cells and tumor microenvironment (such as hypoxia). Collectively, although the mechanisms of intratumor heterogeneity remain unclear and need further study, the exploration of the characteristics of intratumor DNA methylation heterogeneity and the changes of molecular level affected by tumor microenvironment will be helpful for accurately judging tumor properties and finding effective and long-lasting treatment to improve patients' quality of life.

## Conclusions

Combining epigenetic alleles with RRBS permits the identification and characterization of the complexity DNA methylation patterns between tumor core and tumor periphery in breast cancer. Our study reveals a signature of five hypoxia-associated CPDEs that can predict the prognosis of breast cancer patients, which provides guidance for developing effective treatment schemes to improve survival time and quality of patients. As the potential reversibility of DNA methylation, their changes are theoretically amenable to intervention and can be as a promising feature to optimize for devising novel therapeutic targets.

## Supplementary Material

Supplementary figures and tables.

## Figures and Tables

**Table 1:** Univariate and multivariate cox regression in the progress-free survival analysis.

Variable	Univariate analysis			Multivariate analysis		
	HR(95%CI)	Regression coefficient	p	HR(95%CI)	Regression coefficient	p
cg15190451	0.266(0.075-0.947)	-1.324	0.041	0.156(0.04-0.605)	-1.857	0.007
cg08120511	0.204(0.046-0.896)	-1.59	0.035	0.0188(0.039-0.919)	-1.669	0.039
cg27413290	0.088(0.013-0.611)	-2.435	0.014	0. 1(0.015-0.664)	-2.307	0.017
cg10169763	0.002(5.7e-6-0.856)	-6.113	0.044	0.004(2.3e-5-0.808)	-5.451	0.041
cg15891447	0.064(0.007-0.588)	-2.753	0.015	0.081(0.009-0.744)	-2.51	0.026

## Abbreviations

CPDEs: differential epialleles between tumor core and tumor periphery
PPDEs: differential epialleles between tumor periphery and tumor periphery
