# Supplementary material for: Intratumor heterogeneity of breast cancer detected by epialleles shows association with hypoxic microenvironment
Source: Theranostics. 2021 Mar 4;11(9):4403–20. doi: 10.7150/thno.53737 (PMC7977462; doi:10.7150/thno.53737)
Supplement: Supplementary file 1 — Supplementary figures and tables. [file thnov11p4403s1.pdf]

Intratumoral heterogeneity of breast cancer detected by epialleles shows association with hypoxic microenvironment

Yihan Wang[2], Yan Zhang[1,3]✉, Yan Huang[3], Chuangeng Chen[3], Xingda Zhang[4], Ying Xing[5], Yue Gu[3], Mengyan Zhang[3], Li Cai[5], Shouping Xu[4]✉, Baoqing Sun[1]✉

[1] State Key Laboratory of Respiratory Disease, Guangzhou Medical University, Guangzhou, 51000, China

[2] College of Bioinformatics Science and Technology, Harbin Medical University, Harbin, 150081, China.

[3] School of Life Science and Technology, Computational Biology Research Center, Harbin Institute of Technology, Harbin, Heilongjiang 150001, China

[4] Department of Breast Surgery, Harbin Medical University Cancer Hospital, Harbin, Heilongjiang 150081, China.

[5] The Fourth Department of Medical Oncology, Harbin Medical University Cancer Hospital, 150 Haping Road, Harbin, 150040, China

✉Corresponding authors: Yan Zhang, Email: zhangtyo@hit.edu.cn; Baoqing Sun, Email: sunbaoqing@vip.163.com; Shouping Xu, Email：shoupingxu@hrbmu.edu.cn

Supplementary Figures

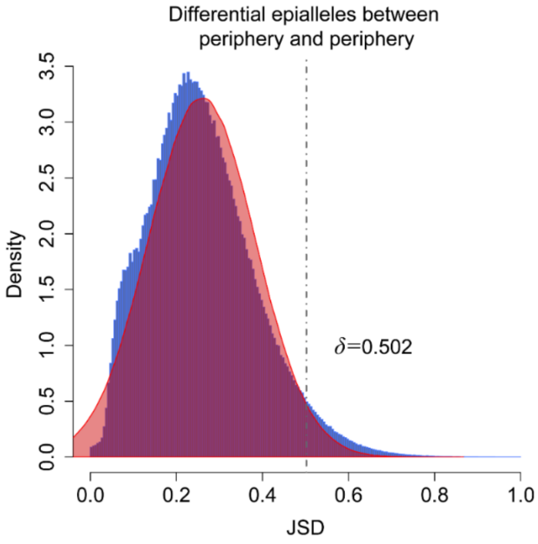

Figure S1 The distribution of JSD and the determination of threshold for identifying differential epialleles between tumor periphery and tumor periphery.

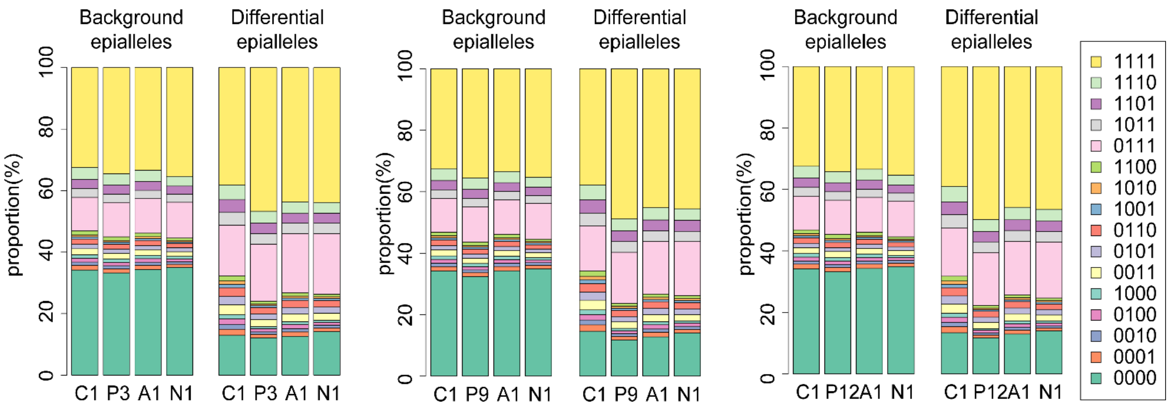

Figure S2 The compositional changes of CPDEs identified between C1 and P3, P9, P12.

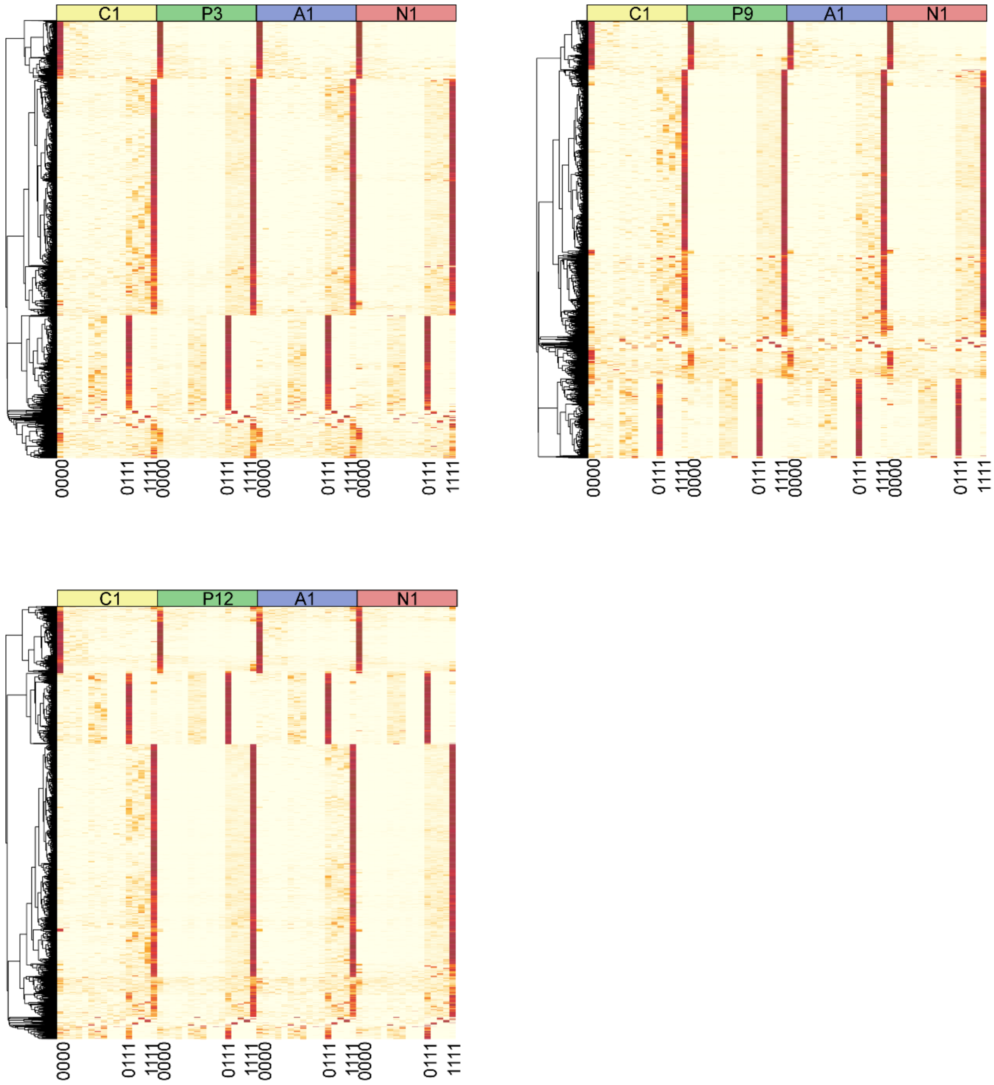

Figure S3 The compositional changes in the core sample (C1), periphery sample (P3/P9/P12), adjacent sample (A1) and normal sample (N1) for the corresponding CPDEs.

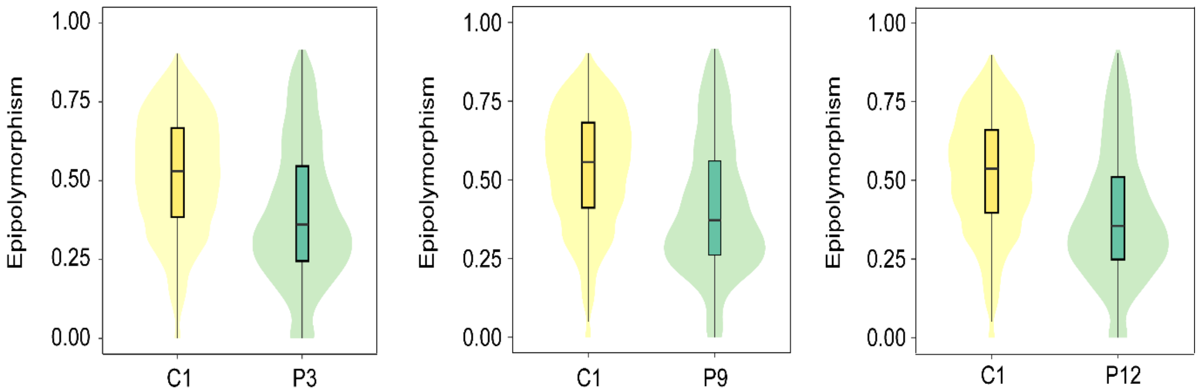

Figure S4 The epipolymorphism in core sample (C1) and periphery samples (P3/P9/P12) for the corresponding CPDEs (Wilcoxon test, $p<0.01$) .

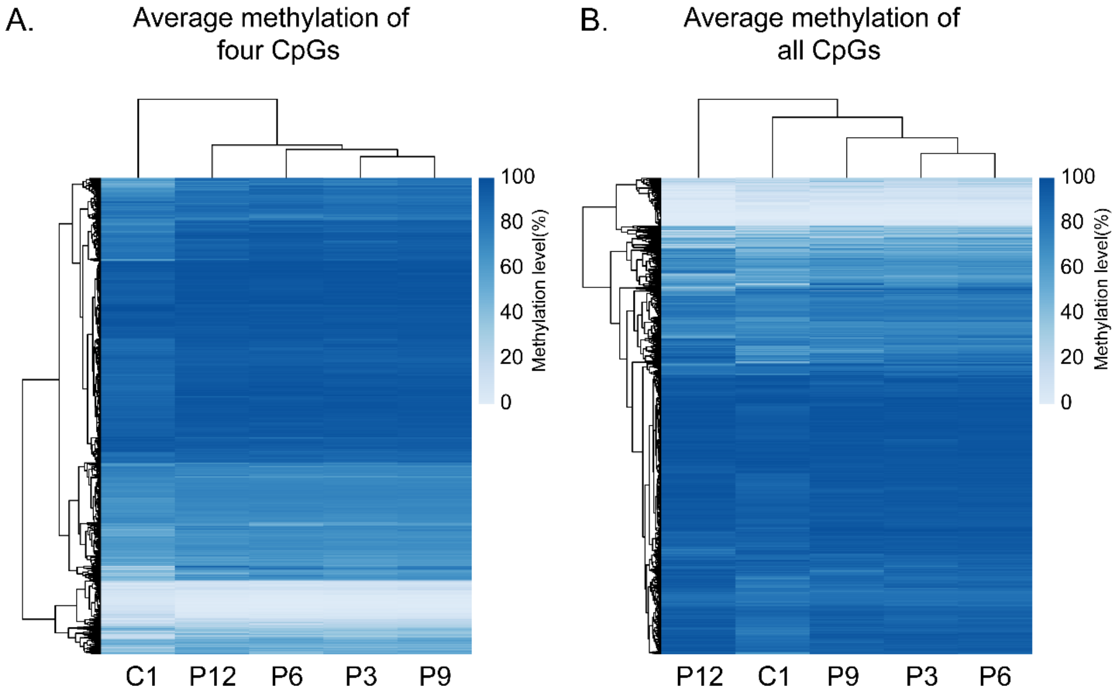

Figure S5 Comparison of two methods for calculating methylation level of epialleles. (A)The heatmap of CPDEs based on average methylation of four CpG sites within the epiallele. (B)The heatmap of CPDEs based on average methylation of all CpG sites within the epiallele.

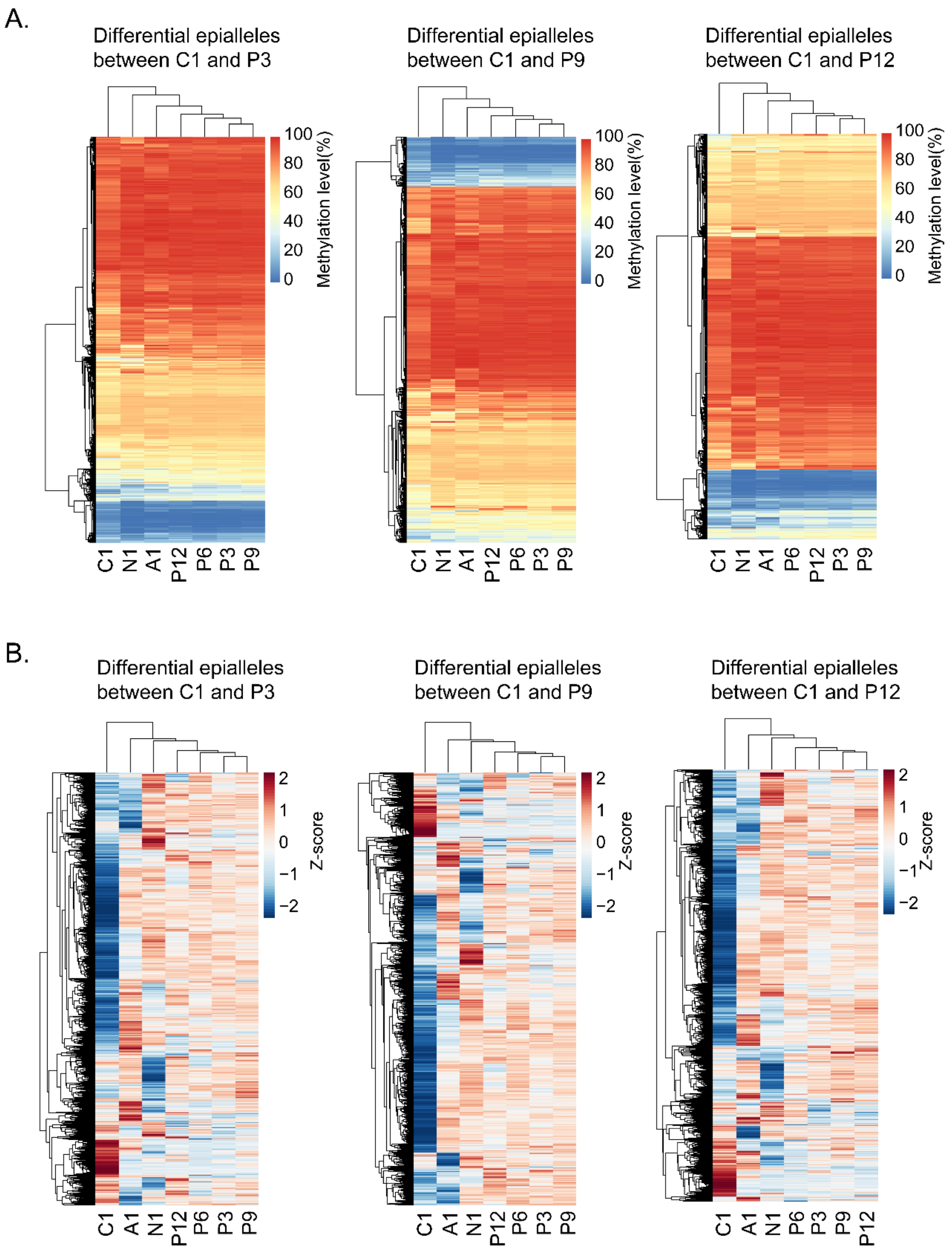

Figure S6 The heatmaps of differential epialleles. (A) The heatmaps of raw methylation level for CPDEs in C1P3, C1P9 and C1P12. (B)The heatmaps of Z-score methylation level for CPDEs in C1P3, C1P9 and C1P12.

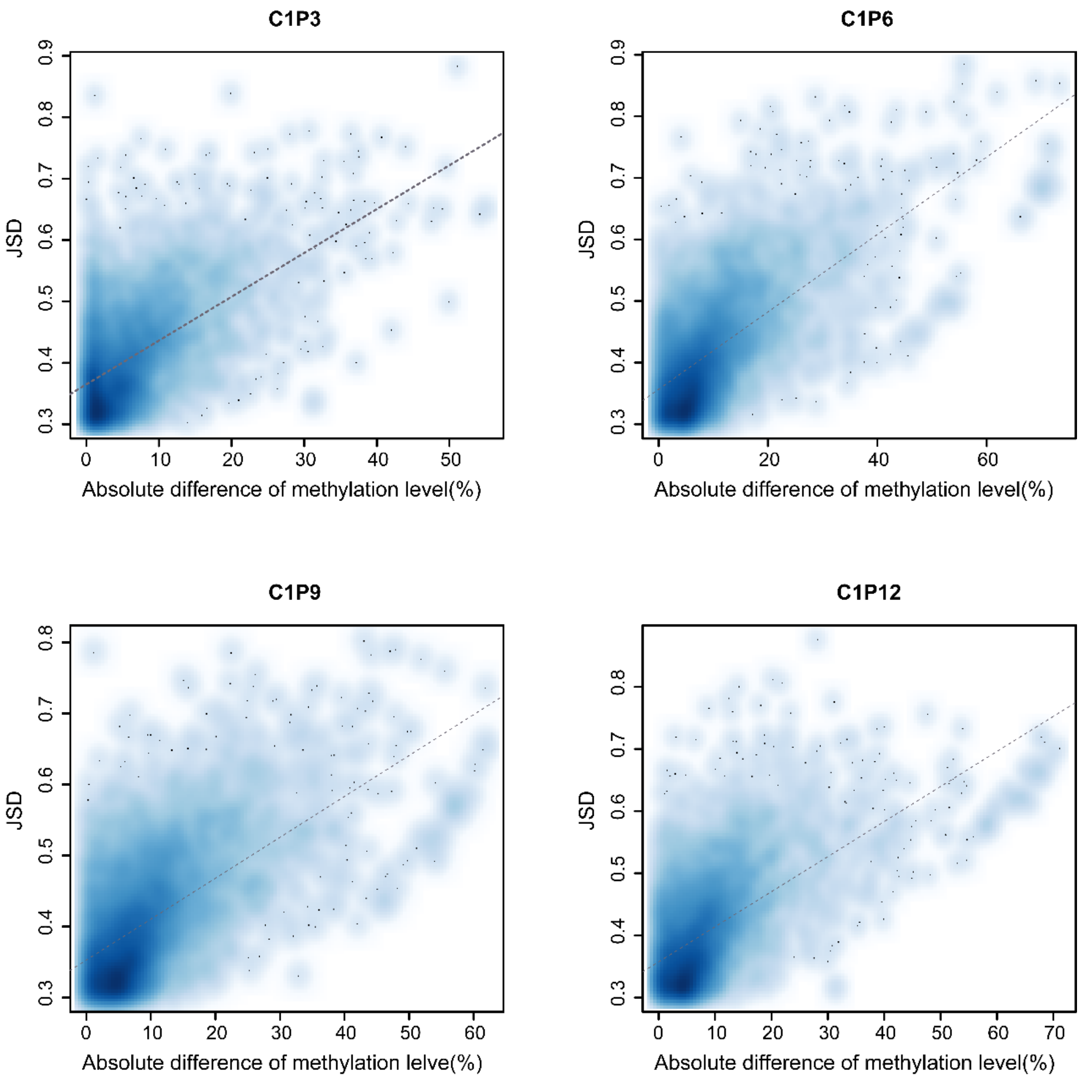

Figure S7 The correlation between JSD and absolute differences of methylation level.

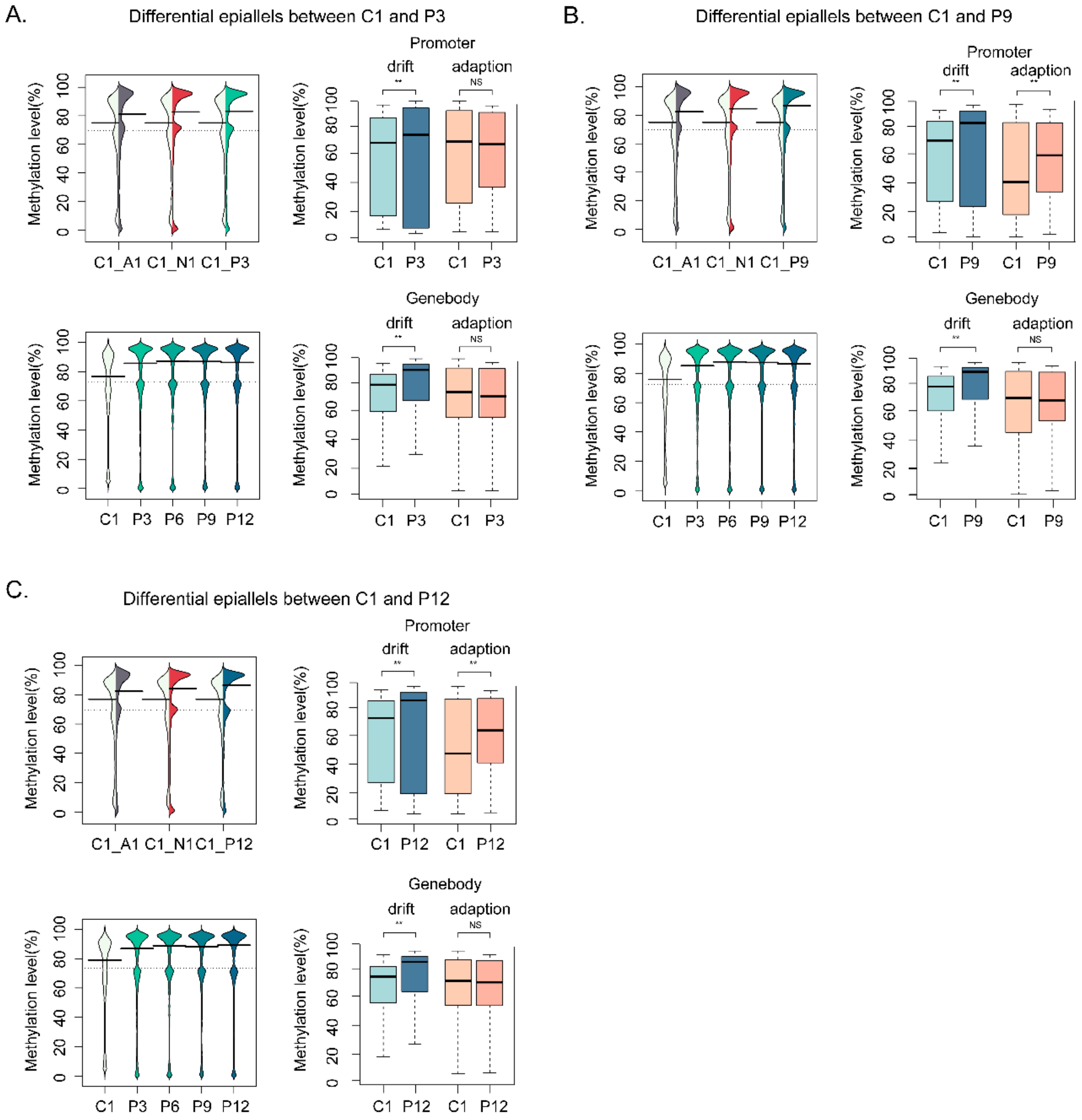

Figure S8 The methylation-based analysis for CPDEs.

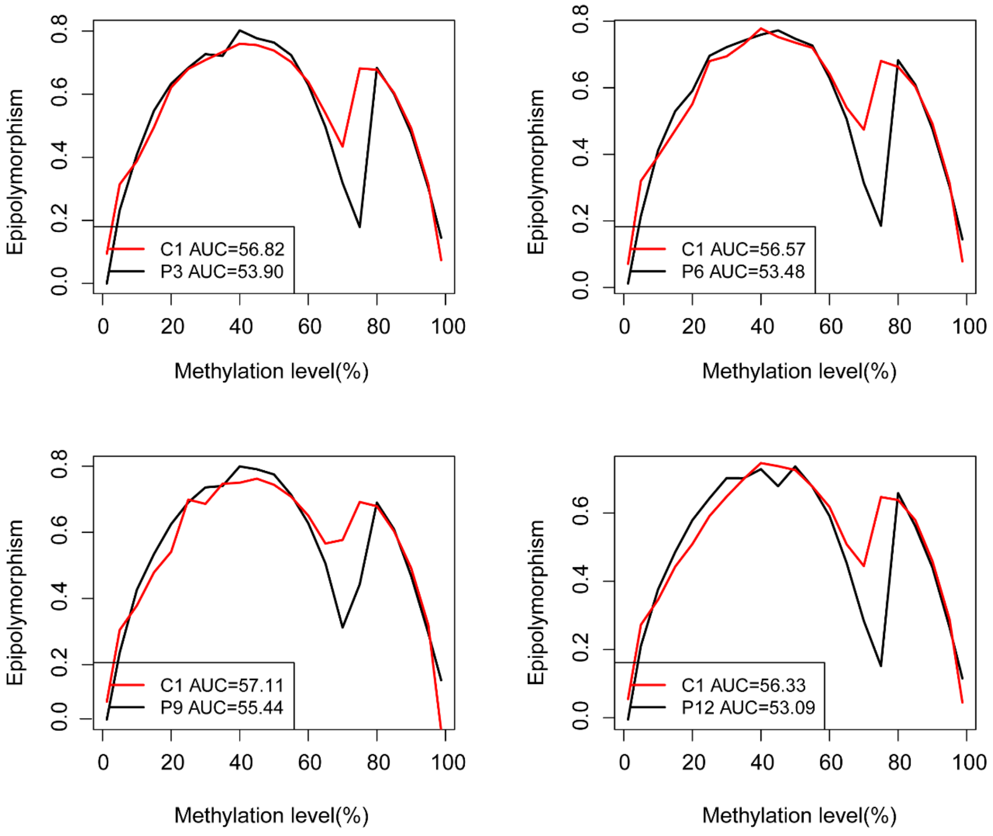

Figure S9 The AUC of methylation heterogeneity in the core sample and the periphery samples.

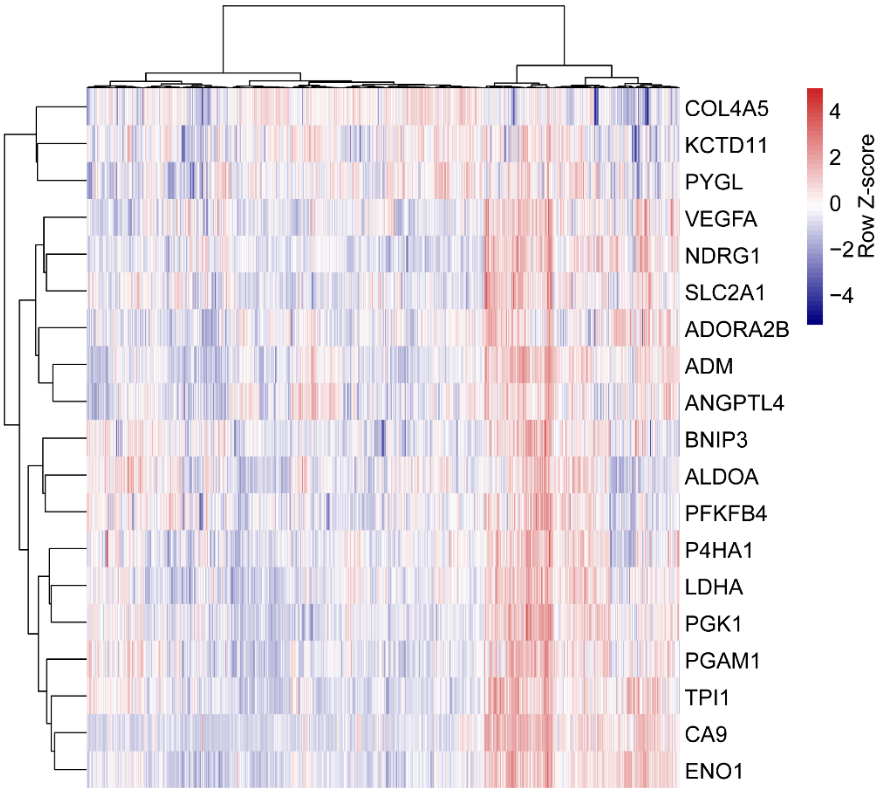

Figure S10 The hierarchical clustering for TCGA BRCA patients based on gene expression of hypoxia signatures.

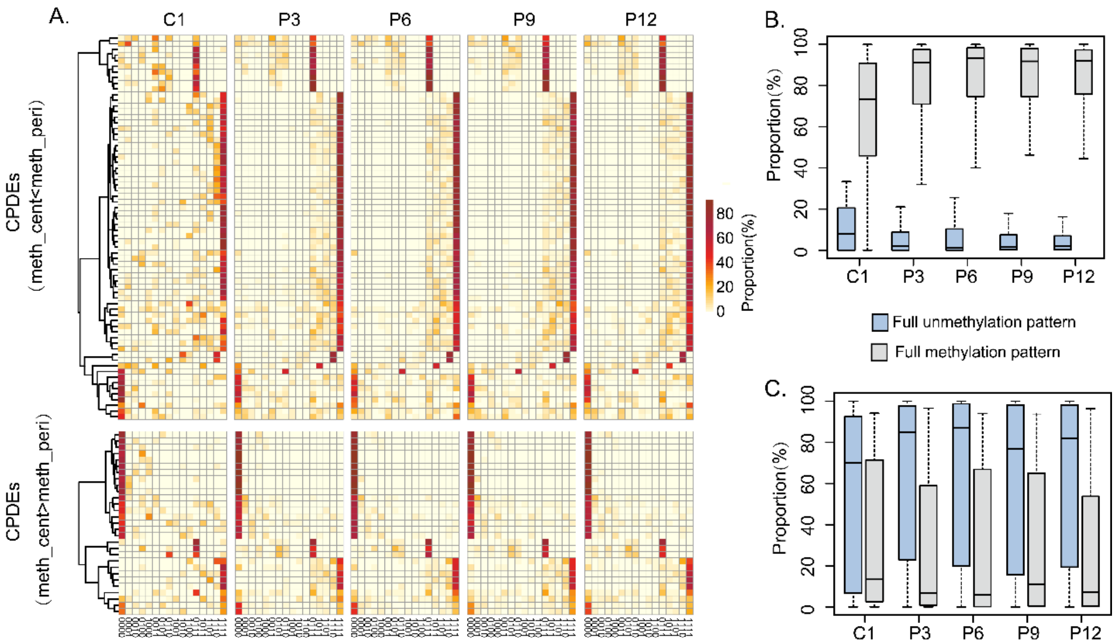

Figure S11 The methylation patterns of 97 CPDEs mapped to 450K CpG probes. (A) The heatmap of methylation pattern of 68 CPDEs hypomethylated in tumor core than periphery, and 29 CPDEs hypermethylated in tumor core than periphery. (B) The proportions of full unmethylation pattern ('0000') and full methylation pattern ('1111') in the two classes of CPDEs.

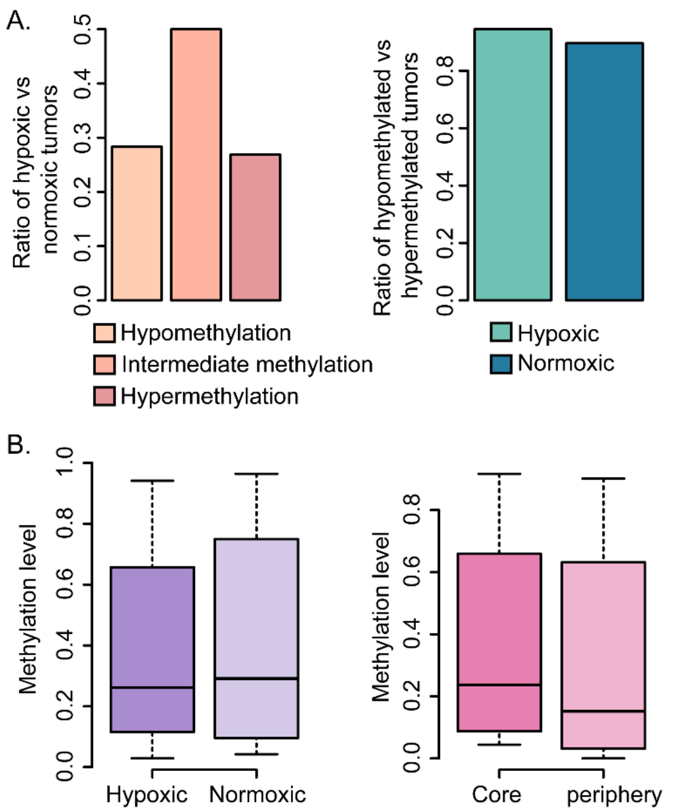

Figure S12 Effect of hypoxia on methylation in CPDEs with lower methylation in tumor core than tumor periphery. (A) Ratios of hypoxic tumors vs normoxic tumors in the hypomethylation, intermediate methylation and hypermethylation groups (Cochran-Armitage trend chi-square test, p=0.805), and ratios of hypomethylated tumors vs hypermethylated tumor in the hypoxic and normoxic clusters (chi-square test, p=1). (B) Boxplots of methylation level in hypoxic and normoxic clusters, as well as tumor core and tumor periphery.

Supplementary Tables

Table S1 The information of RRBS data for seven samples.

Sample	C1	P3	P6	P9	P12	A1	N1
Raw reads	58,822,682	56,023,350	50,470,280	109,323,286	141,953,654	68,383,106	56,725,878
Clean reads	57,623,782	42,174,314	42,173,590	95,971,184	86,778,070	67,195,330	52,397,042
Depth	18.41727	18.85564	22.8961	29.95219	43.93936	20.03477	21.15825
CpGs	9,134,184	7,318,198	6,480,995	10,978,312	7,981,636	9,754,366	8,019,834
Mapping efficiency	74.9%	72.4%	71.5%	76.7%	73.4%	71.0%	75.2%

Table S2 The information of RNA-seq data for seven samples.

Sample	C1	P3	P6	P9	P12	A1	N1
Reads	40,407,794	63,768,476	90,734,586	73,727,062	53,847,362	46,418,336	43,473,922
Mapping Rate	84%	81.6%	66.3%	72.2%	82.5%	87.7%	89.1%

Table S3 The number of each class of TCGA BRCA samples.

	Hypoxic	Normoxic
Hypermethylation	32	133
Intermediate methylation	77	197
Hypomethylation	100	145

Table S4 The CPDEs with lower methylation level in tumor core than tumor periphery.

chr	start	end	epiallele	CpG
16	85485722	85485786	85485722:85485745:85485764:85485786	cg05905731
2	128350569	128350595	128350569:128350571:128350589:128350595	cg01577298
X	153657332	153657363	153657332:153657335:153657357:153657363	cg04334684
15	67073416	67073442	67073416:67073426:67073433:67073442	cg03925327
13	114004028	114004140	114004028:114004039:114004072:114004140	cg15828353
X	153657335	153657412	153657335:153657357:153657363:153657412	cg04334684
X	153657335	153657412	153657335:153657357:153657363:153657412	cg05872808
11	78387281	78387323	78387281:78387291:78387311:78387323	cg15006118
5	149546074	149546104	149546074:149546088:149546099:149546104	cg25132276
11	719705	719729	719705:719711:719713:719729	cg17469934
10	1205650	1205691	1205650:1205652:1205668:1205691	cg15057747
17	77901103	77901148	77901103:77901130:77901144:77901148	cg11339839
20	49621249	49621314	49621249:49621297:49621306:49621314	cg04524933
17	73096446	73096503	73096446:73096456:73096476:73096503	cg21853806
17	73096446	73096503	73096446:73096456:73096476:73096503	cg24328816
6	168686236	168686300	168686236:168686247:168686270:168686300	cg05571645
6	168686236	168686300	168686236:168686247:168686270:168686300	cg21701043
22	30950920	30950958	30950920:30950930:30950934:30950958	cg22934785
1	2108696	2108749	2108696:2108706:2108730:2108749	cg02334669
8	144552717	144552771	144552717:144552730:144552736:144552771	cg27413290
2	241827847	241827876	241827847:241827852:241827864:241827876	cg24075745
10	1205538	1205572	1205538:1205563:1205565:1205572	cg09426810
10	1205538	1205572	1205538:1205563:1205565:1205572	cg26293457
2	75720531	75720567	75720531:75720540:75720546:75720567	cg00409636
7	813677	813751	813677:813704:813709:813751	cg26465987
7	813704	813764	813704:813709:813751:813764	cg26465987
20	60969897	60969964	60969897:60969906:60969929:60969964	cg23531748
5	2749647	2749715	2749647:2749649:2749655:2749715	cg07511080
12	132834295	132834340	132834295:132834315:132834336:132834340	cg04774461
4	24801110	24801131	24801110:24801120:24801125:24801131	cg10766585
21	15135117	15135146	15135117:15135134:15135142:15135146	cg11939992
16	30616268	30616334	30616268:30616299:30616309:30616334	cg05045702
16	88738398	88738418	88738398:88738410:88738413:88738418	cg03682568
16	88738398	88738418	88738398:88738410:88738413:88738418	cg04176488
12	131519904	131519996	131519904:131519927:131519993:131519996	cg13586425
16	88018664	88018712	88018664:88018667:88018681:88018712	cg26605774
20	60969885	60969929	60969885:60969897:60969906:60969929	cg23531748
6	168686216	168686270	168686216:168686236:168686247:168686270	cg21701043
X	132550050	132550132	132550050:132550080:132550086:132550132	cg12600766
19	7677731	7677748	7677731:7677733:7677740:7677748	cg02076378
22	30950923	30950947	30950923:30950930:30950938:30950947	cg22934785
19	4544068	4544095	4544068:4544080:4544082:4544095	cg04848343

18	34340652	34340700	34340652:34340681:34340691:34340700	cg23695714
19	7584666	7584698	7584666:7584674:7584680:7584698	cg01773515
1	161576928	161576989	161576928:161576958:161576964:161576989	cg26022242
13	19412134	19412226	19412134:19412185:19412222:19412226	cg08120511
7	23695224	23695277	23695224:23695247:23695263:23695277	cg15781397
17	73090153	73090195	73090153:73090163:73090186:73090195	cg15190451
20	49626332	49626374	49626332:49626346:49626348:49626374	cg03711791
X	136648305	136648319	136648305:136648308:136648315:136648319	cg22934939
11	67261122	67261175	67261122:67261166:67261172:67261175	cg17028218
X	100306753	100306832	100306753:100306781:100306813:100306832	cg13286902
X	100306753	100306832	100306753:100306781:100306813:100306832	cg15891447
11	490498	490558	490498:490525:490552:490558	cg11004323
16	87873347	87873423	87873347:87873354:87873369:87873423	cg10169763
18	34340640	34340681	34340640:34340642:34340652:34340681	cg23695714
22	30950930	30950961	30950930:30950934:30950958:30950961	cg22934785
2	242608073	242608094	242608073:242608075:242608087:242608094	cg06006530
2	242608073	242608094	242608073:242608075:242608087:242608094	cg08462942
17	62066571	62066673	62066571:62066580:62066597:62066673	cg08387141
15	40751007	40751064	40751007:40751013:40751016:40751064	cg02479871
19	39217718	39217766	39217718:39217722:39217738:39217766	cg25383568
6	168686247	168686309	168686247:168686270:168686300:168686309	cg05571645
20	36532087	36532097	36532087:36532089:36532091:36532097	cg11599505
1	5934887	5934950	5934887:5934913:5934941:5934950	cg09976670
3	114070427	114070444	114070427:114070434:114070441:114070444	cg19622597
1	2108706	2108769	2108706:2108730:2108749:2108769	cg02334669
17	77901150	77901227	77901150:77901179:77901224:77901227	cg05483571
7	98609840	98609873	98609840:98609846:98609859:98609873	cg04212699
12	131519890	131519927	131519890:131519899:131519904:131519927	cg13586425
11	68170975	68171011	68170975:68170999:68171003:68171011	cg25380755
2	242608075	242608104	242608075:242608087:242608094:242608104	cg06006530
2	242608075	242608104	242608075:242608087:242608094:242608104	cg08462942
19	4544080	4544105	4544080:4544082:4544095:4544105	cg04848343
16	88018667	88018719	88018667:88018681:88018712:88018719	cg26605774
16	10274064	10274089	10274064:10274070:10274086:10274089	cg10116505
